# What are the benefits of directed attention within verbal working memory?

**DOI:** 10.1177/17470218241299918

**Published:** 2024-12-10

**Authors:** Stéphanie Jeanneret, Evie Vergauwe, Caro Hautekiet, Naomi Langerock

**Affiliations:** Faculté de Psychologie et des Sciences de l’Éducation, Université de Genève, Genève, Switzerland

**Keywords:** Verbal working memory, attention, focus of attention, distraction, vulnerability, memory boost

## Abstract

Information that is particularly relevant for upcoming behaviour can be prioritised within working memory, by directing attention to it. Receiving focused attention during retention is assumed to be associated with specific benefits, such as increased memory performance and reduced vulnerability to perceptual distractions. This has been demonstrated in visuospatial working memory. Given the domain-general nature of the focus of attention, these benefits should extend to verbal working memory as well. This was tested in the current study. In particular, we examined and compared the effects of cue-based and reward-based prioritisation in verbal working memory across a series of five preregistered experiments. These experiments varied in their memory materials, set size, interference, and memory task. Our results collectively revealed several key findings. First, both cue-based and reward-based prioritisation led to a clear and consistent memory boost for prioritised information in verbal working memory. Second, the memory boost induced by cue-based prioritisation was mostly comparable to that induced by reward-based prioritisation. Third, memory for verbal information did not drastically suffer when exposed to perceptual interference. And finally, the effect of perceptual interference on verbal information was not drastically influenced by whether the information was prioritised or not. Overall, this series of experiments contributes to understanding the consequences of directed attention in verbal working memory and highlights similarities and differences from findings in visuospatial working memory.

The cognitive system that holds information accessible and available in mind is referred to as working memory. Within working memory, attention can be directed to a subset of the represented information such that it can serve as the object of the upcoming cognitive action or operation. The subset of information that receives focused attention is said to be *prioritised* within working memory. During retention, being held in this state is assumed to be associated with specific benefits such as increased memory performance and reduced distractor susceptibility. Although the focus of attention within working memory is typically assumed to be domain-general in nature, the benefits of directed attention within working memory have primarily been studied in the visuospatial domain of working memory. In the current study, we aimed to assess the consequences of directed attention in the verbal domain of working memory.

## The consequences of directed attention in the visuospatial domain of working memory

In visuospatial working memory, the consequences of directed attention have traditionally been studied using the retrospective cue (“retro-cue”) paradigm (Griffin & Nobre, 2003; Landman et al., 2003). In this paradigm, encoding is followed by a retention interval during which a cue can be presented. This cue signals which memory item is most likely to be tested after the retention interval, thus indicating which memory item is most relevant for the current trial. Often, the cue is spatial, indicating the location where the most relevant memory item was presented during encoding. The assumption is that the cue directs the focus of attention within working memory to the representation of the item that had been displayed at the cued location, thereby prioritising the cued item in working memory. Memory performance is usually better for the cued item than for uncued items, which come either from trials without a retro-cue or from neutral cue trials providing no information about which item is most likely to be tested (see Souza & Oberauer, 2016, for a review). This memory boost for cued information has been observed in many studies (e.g., Barth & Schneider, 2018; Gunseli et al., 2015; Hautekiet et al., 2023; Jeanneret et al., 2023; Lepsien & Nobre, 2007; Makovski & Pertzov, 2015; Schneider et al., 2017; Sligte et al., 2008; van Moorselaar et al., 2015; Vergauwe et al., 2023, in preparation; Zhang & Lewis-Peacock, 2023a, 2023b) and is regarded as one of the main benefits of bringing information into the focus of attention during memory retention.

A second benefit often associated with directed attention during working memory retention is reduced distractor susceptibility. This effect has been demonstrated in studies where irrelevant information is presented between cue and test. Here, memory performance for cued items is found to suffer *less* from the presence of perceptual interference than memory performance for uncued items does (e.g., Barth & Schneider, 2018; Makovski & Jiang, 2007; Schneider et al., 2017; Souza et al., 2016; van Moorselaar et al., 2015; Vergauwe et al., 2023; but see Hautekiet et al., 2023). The observation that cued information appears protected from perceptual interference, compared to uncued information, mirrors the theoretical notion that the focus of attention is a privileged state within working memory where information enjoys protection from interference (e.g., Cowan, 2011; Jonides et al., 2008; McElree, 2006; Oberauer, 2002, 2009; Oberauer & Lin, 2017; Pertzov et al., 2013; Souza & Oberauer, 2016).

Although the use of retro-cues has been the most popular way to direct attention during working memory retention in visuospatial working memory, other prioritisation modes have been used. One of these alternatives is the use of reward patterns. Whereas cue-based prioritisation uses cues to indicate which item is most likely to be tested, reward-based prioritisation uses reward values to indicate which item is associated with a high reward value to direct the focus of attention within working memory (e.g., Allen & Ueno, 2018; Hu et al., 2014). In some studies, reward values are associated with the memory items by presenting a spatial reward pattern signalling the number of points one can gain if the memory item presented in that location is correctly recalled or recognised at the end of the trial. This has been done by presenting a pattern of digits, with one digit shown in each location where a memory item can appear, such as a “4” in the location of the high-reward item and a “1” in the other locations (e.g., Allen & Atkinson, 2021; Allen & Ueno, 2018; Atkinson et al., 2022; Jeanneret et al., 2023; Zhang & Lewis-Peacock, 2023b), or by highlighting one location using a bolded frame at the location of the high-reward item while leaving the frames at the other locations unbolded (e.g., Hautekiet et al., 2024; Vergauwe et al., 2023, in preparation). These reward patterns have been presented both before encoding (e.g., Allen & Atkinson, 2021; Allen & Ueno, 2018; Atkinson et al., 2022; Vergauwe et al., 2023) and after encoding (Allen & Atkinson, 2021; Hautekiet et al., 2024; Jeanneret et al., 2023; Vergauwe et al., 2023, in preparation). In other studies, reward values are associated with the visuospatial memory items by providing specific instructions that associate different reward values with different serial positions and that are given before encoding (e.g., more points can be gained if the item presented in the first serial position is correctly recalled; e.g., Atkinson et al., 2018; Hitch et al., 2018; Hu et al., 2014, 2016).

Across these various reward manipulations, memory performance is usually observed to be better for the high-reward item than for low-reward items or equal-reward items coming from trials without a reward pattern (e.g., Allen & Atkinson, 2021; Allen & Ueno, 2018; Atkinson et al., 2018, 2022; Hautekiet et al., 2024; Hitch et al., 2018; Hu et al., 2014, 2016; Jeanneret et al., 2023; Vergauwe et al., 2023). However, studies having directly compared retrospective cue-based and reward-based prioritisation in visuospatial working memory often found the memory boost due to reward-based prioritisation to be smaller and less consistent than the memory boost due to cue-based prioritisation (Hautekiet et al., 2024; Jeanneret et al., 2023; Vergauwe et al., 2023, in preparation; Zhang & Lewis-Peacock, 2023b). For example, Jeanneret et al. (2023) found evidence for a memory boost with cue-based prioritisation during retention in two experiments, while evidence for a memory boost using reward-based prioritisation was only observed in one of these experiments. Similarly, Vergauwe et al. (2023) observed a much larger memory boost with cue-based prioritisation during retention than with reward-based prioritisation (both when comparing high-reward items to low-reward items and when comparing high-reward items to equal-reward items).

As mentioned previously, another benefit of directed attention within working memory is that of reduced distractor susceptibility. However, to our knowledge, no evidence has been reported for reduced distractor susceptibility for prioritised visuospatial information with reward-based prioritisation. Instead, studies presenting perceptual interference either have observed that high-reward items suffer just as much from perceptual interference as the other items do (e.g., Allen & Ueno, 2018; Hu et al., 2014; Vergauwe et al., 2023) or have found that high-reward items suffer *more* from perceptual interference than other items do (e.g., Allen & Ueno, 2018; Hitch et al., 2018; Hu et al., 2014, 2016). Thus, with regards to reward-based prioritisation, evidence for a protected state in the focus of attention seems to be lacking in visuospatial working memory. Importantly, the absence of reduced distractor susceptibility for high-reward items has also been observed within the same paradigm that *did* show reduced distractor susceptibility for cued items (Vergauwe et al., 2023), thereby eliminating several methodological differences between cue-based and reward-based conditions.

Overall, the general understanding concerning the consequences of directed attention in visuospatial working memory emerging across retrospective cue-based and reward-based prioritisation studies is that (1) a memory boost is usually observed for prioritised information using both prioritisation modes, although the boost is often less consistent and smaller with reward-based prioritisation, and (2) reduced distractor susceptibility is usually observed for prioritised information with cue-based prioritisation but not with reward-based prioritisation. Given that the observed pattern in visuospatial working memory seems to diverge to a certain extent between the two prioritisation modes that are frequently used in the field, it appears that understanding the consequences of directed attention may be less straightforward than previously thought. Moreover, one should not rely on the observations under a single prioritisation mode when studying the consequences of directed attention in working memory.

## The consequences of directed attention in the verbal domain of working memory

The focus of attention in working memory has typically been assumed to be domain-general in nature (e.g., Baddeley et al., 2021; Barrouillet et al., 2007; Cowan, 2005; Oberauer, 2002). Within the framework of the multiple-component model, the prioritisation of information within working memory through the use of the focus of attention is assumed to be closely related to the domain-general components of working memory as well, which Hitch and Baddeley (1976) referred to as “central WM executive” (e.g., see Hitch et al., 2018, 2020; Hu et al., 2014, for papers linking attention-based prioritisation to the central executive, and e.g., see Atkinson et al., 2021; Baddeley et al., 2021, for papers linking the focus of attention to the episodic buffer). Therefore, the benefits of information being held in the focus of attention during retention should reasonably extend to the different domains of working memory. However, as previously mentioned, the consequences of directed attention on working memory representations have predominantly been studied using visuospatial materials. Therefore, it is currently unclear whether the same pattern of consequences across different prioritisation modes exists in verbal working memory. If this is the case, it would require models of working memory to account for domain-general aspects of attention in working memory while explaining variations across prioritisation modes. Finding the same pattern in verbal working memory would reinforce the previous observations in visuospatial working memory, which would (1) strongly suggest the operation of a domain-general focus of attention in working memory operating similarly across different types of information and (2) emphasise the need to revise existing models to accommodate the varying consequences of directed attention across different prioritisation modes. If, on the other hand, the pattern in verbal working memory diverges from that observed in visuospatial working memory, models would have to account for not only the fact that the consequences of directed attention vary across prioritisation modes but also the fact that these consequences vary across domains.

To our knowledge, only a few studies have investigated the consequences of directed attention in working memory using verbal materials. Some of these studies have used specific maintenance instructions whereby participants are asked to think back to verbal memory items (letters, words, or word pairs) during retention, a maintenance strategy referred to as refreshing and assumed to rely on the focus of attention (see Camos et al., 2018, for a review). While one study found some evidence for a memory boost associated with refreshing of verbal materials (Souza et al., 2018), others did not (Bartsch et al., 2018; Vergauwe, 2018). More direct manipulations of the focus of attention were used in studies that relied on cue-based or reward-based prioritisation to direct attention during working memory retention of verbal materials. While evidence suggests a memory boost for both prioritisation modes, no study has directly compared these memory boosts, leaving it unclear whether, as in visuospatial working memory, the boost for verbal materials tends to be smaller and less consistent with reward-based than with cue-based prioritisation.

As far as cue-based prioritisation is concerned, Krefeld-Schwalb (2018) presented a visual array of 4–8 letters on each trial, which could be followed by a retro-cue pointing to one of the locations where a letter had been presented. Although the short presentation duration of the array (1000 ms, i.e., only between 125 and 250 ms per letter) may have encouraged visual encoding of these letters, especially at larger set sizes, the observation of better memory performance for cued letters relative to uncued letters (from trials without a cue) suggests that directed attention during retention may also boost memory for verbal memory materials (see also the article by Sligte et al., 2008, for a study with similar findings, using arrays of alphanumeric characters with an even shorter presentation duration of 250 ms in total for eight characters, i.e., about 30 ms per letter). Shepherdson et al. (2018) also examined the retro-cue effect with verbal materials and observed a memory boost for cued letters that had been presented simultaneously in an array (relative to trials without a cue), again using a rather short presentation duration (150 ms per letter), but not for cued words that had been presented sequentially in a list (at a rate of 500 ms per word). This could indicate that the evidence for a memory boost in verbal working memory may be less consistent than in visuospatial working memory when using cue-based prioritisation.

The few studies using reward-based prioritisation of verbal materials have consistently shown a memory boost. These studies used series of digits, letters, or words (presented at rates of 500 ms per item, or slower) and manipulated reward values either by associating different reward values with serial positions before encoding (Atkinson et al., 2021) or by presenting one of the memory items in a specific colour (Sandry et al., 2014, 2020). In all cases, high-reward items outperformed items at the same serial position on trials without a reward manipulation. Thus, when using reward-based prioritisation, directing attention to verbal material during retention has resulted in a memory boost. However, no study has used retrospective reward-based prioritisation of verbal memory materials, and thus, it is not known whether verbal information can benefit from being associated with a high reward after encoding.

The second benefit associated with directed attention during working memory retention, that of reduced distractor susceptibility, has not been studied yet in verbal working memory. To our knowledge, only one study focused on how distraction affects prioritized vs. unprioritized information in verbal working memory. In that study, Krefeld-Schwalb (2018) used cue-based prioritisation and introduced three secondary tasks following the cue. All tasks were active tasks that required comparison, decision, and response-selection processes. In two out of the three tasks, evidence was found for protected cued information relative to uncued information. The task that may be considered the most similar to the perceptual interference conditions that are typically used in visuospatial working memory experiments to examine distractor susceptibility of prioritised information did not show reduced distractor susceptibility (i.e., the *perception* task in their Experiment 1b).^1^ Importantly, however, in that condition, there was no negative impact of the presence of the interfering task at all, neither for the cued items, nor for the uncued items. Thus, based on the currently available data, we cannot infer whether directed attention during retention in verbal working memory is associated with reduced distractor susceptibility, nor whether the pattern would vary between cue-based and reward-based prioritisation. The current study aims to fill these gaps in our knowledge.

## The current study

The goal of the current study was to assess the consequences of directed attention in verbal working memory. In particular, we aimed to examine to what extent prioritised verbal information enjoys the assumed benefits of being held in the focus of attention during working memory retention: a memory boost and reduced distractor susceptibility. Since the findings in visuospatial working memory have indicated that the consequences of directed attention may differ depending on how the focus of attention is directed to a specific representation within working memory, we decided to use both cue-based and reward-based prioritisation in the current study. Moreover, to allow for a direct comparison of the findings across prioritisation modes, we decided to take a similar approach as the one reported in Vergauwe et al. (2023), where the impact of cue-based and reward-based prioritisation was investigated within a single paradigm in which as many methodological variables were kept constant across the prioritisation modes as possible (e.g., the priority signal, nature of memory materials, number of memory items, presentation times, nature of perceptual interference, and the type of memory test). As explained in the introduction, it is clear that many methodological factors have widely varied among studies using cue-based vs. studies using reward-based prioritisation in verbal working memory. Since, in a next step, we also wanted to compare our new findings in verbal working memory with those reported in visuospatial working memory, especially those reported in Vergauwe et al. (2023), we decided to create a verbal analogue of the visuospatial paradigm used in Vergauwe et al. (2023) for our first experiment. This implied that we used arrays of verbal materials (like most cue-based studies, but unlike reward-based studies in verbal working memory, which used sequential presentation). Importantly, however, we presented the verbal memory arrays for 400 ms per item, which is longer than the letter arrays in the cue-based studies (Krefeld-Schwalb, 2018; Shepherdson et al., 2018; Sligte et al., 2008) and more in line with the reward-based studies in verbal working memory using letters and words (Atkinson et al., 2021; Sandry et al., 2014, 2020). Finally, whereas previous studies in visuospatial working memory often used fast presentation rates or imposed articulatory suppression to avoid verbal recoding and minimise the involvement of verbal representations and strategies in maintenance (e.g., Allen & Ueno, 2018; Hu et al., 2014; Vergauwe et al., 2023), this was not the case here because we aimed to investigate the impact of directed attention on verbal representations.

Below, we report a series of five experiments. An overview of the methodological differences between the experiments, as well as the outcomes that were assessed, can be found in Table 1. All experiments required maintaining an array of visually-presented words. Experiments 1–3 assessed both memory performance and distractor susceptibility as a function of the Prioritisation Status (prioritized information vs. unprioritized information) and Prioritisation Mode (cue-based vs. reward-based prioritisation). To anticipate, clear memory boosts were found for prioritised information, both with cue-based and reward-based prioritisation. However, in Experiment 1, we could not investigate whether prioritised information enjoys reduced distractor susceptibility because the perceptual interference did not affect memory performance. To follow up on Experiment 1, we designed two additional experiments in which we aimed to assess both memory performance and distractor susceptibility again, while increasing our chances of observing a negative impact of perceptual interference overall. In Experiment 2, we targeted the nature of the perceptual interference and made several modifications to it to make it harder for participants to potentially ignore the distracting information during retention. In Experiment 3, we primarily targeted the memory task itself and made several modifications to it to make the memory task more similar to some of the tasks in visuospatial working memory in which a negative impact of perceptual interference was found. To anticipate, while perceptual interference did affect memory performance in Experiments 2 and 3, its impact was far from drastic, and there was not much evidence for any modulation of distractor susceptibility as a function of whether attention had been directed to the information during retention.

**Table 1.** Table reporting experimental factors that could change from one experiment to another in Experiments 1–5: memory materials, set size, prioritisation manipulation, perceptual interference, and memory test, as well as the outcomes that are tested per experiment.

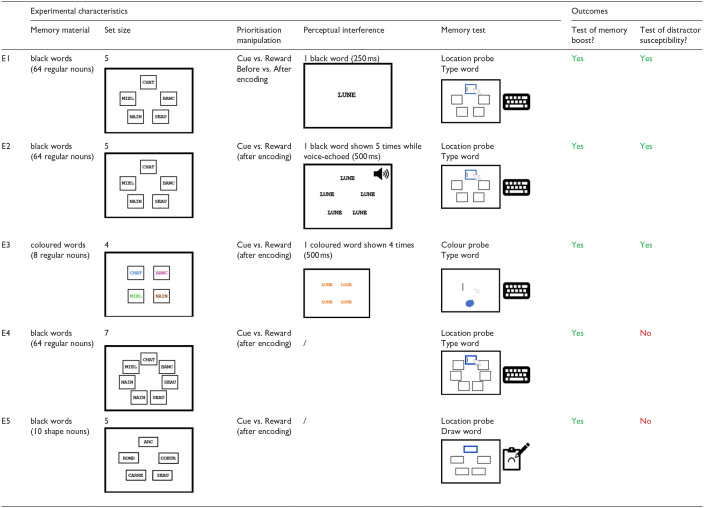

Thus, based on Experiments 1–3, it seemed that information in verbal working memory was quite robust to perceptual interference that is similar to what has been used in visuospatial working memory (even when it was presented auditorily in Experiment 2), and the impact of perceptual interference was remarkably similar across prioritized and unprioritized working memory content. When it comes to the memory boost, the data from Experiments 1–3 showed that both cue-based and reward-based prioritisation resulted in better memory performance for the prioritised information. Moreover, whereas in visuospatial working memory, the memory boost often appeared larger and more consistently for cue-based prioritisation than for reward-based prioritisation, this was not the case in our first three experiments, where a boost was consistently found for both prioritisation modes and where the size of the boosts was similar across cue-based and reward-based prioritisation. Based on this observation, we decided to focus only on the memory boost effect in the final Experiments 4 and 5. In particular, to understand whether the apparent difference between verbal and visuospatial experiments reflects domain-related differences, we explored potential alternative explanations for the difference in the memory boost patterns between verbal and visuospatial experiments.

Overall, Experiments 1–5 collectively show (1) that both retrospective cue-based prioritisation and reward-based prioritisation consistently result in a memory boost for prioritised verbal information; (2) that the memory boost induced by retrospective cue-based prioritisation is neither more consistent nor consistently larger than the memory boost induced by retrospective reward-based prioritisation; (3) that simultaneously presented verbal information does not suffer greatly when perceptual interference that is similar to what has been used in visuospatial working memory is presented during retention, even when presented auditorily; and (4) that the effect of perceptual interference on simultaneously presented verbal information is only minimally impacted by the prioritisation status of the said information. Overall, this pattern of findings shares certain similarities with findings in visuospatial working memory, but it also exhibits important differences. All experiments in this series were preregistered prior to being conducted.

## Experiment 1

Experiment 1 aimed to create a verbal analogue of the visuospatial paradigm used in Experiment 2 of Vergauwe et al. (2023). Cue-based prioritisation studies in verbal working memory have only prioritised information after encoding, whereas reward-based prioritisation studies in verbal working memory have only prioritised information before or during encoding. Therefore, and in line with the work by Vergauwe et al. (2023), we manipulated not only Prioritisation Mode (cue-based vs. reward-based prioritisation) but also the timepoint at which the prioritisation signal was given (pre- or post-encoding), to examine whether the pattern of findings would be modulated as a function of when attention was directed to one specific item.^2^

### Method

The preregistration for Experiment 1 can be found at https://osf.io/snbme.

#### Participants and design

Our sample size was determined by the Bayesian sequential hypothesis testing procedure described in Supplementary Materials 1. A total of 62 undergraduate students (50 female, 12 male, 0 other; mean age = 22 years) were recruited from the University of Geneva and participated in exchange for partial course credit. All participants had normal or corrected-to-normal vision. There were 30 participants in the Cue group and 32 participants in the Reward group. No performance-based exclusions were applied.

We implemented a 2 × 3 × 2 design, with Prioritisation Mode (Cue vs. Reward) as between-subjects variable, and Prioritisation Timepoint (Pre-prioritisation, Post-prioritisation, or No-prioritisation) and Distraction (Suffix vs. No suffix) as within-subjects variables. In each group (Cue and Reward), the Prioritisation Timepoint was implemented in blocks, and the order of the blocks was counterbalanced across participants. In total, participants completed 300 experimental trials. These trials were presented in three blocks of 100 trials, where each block corresponded to a Prioritisation Timepoint. Within each block, *Suffix* and *No-suffix* trials were equally likely and randomly intermixed (i.e., 50 trials per block included a suffix item). Total experiment runtime, including consent and on-screen instructions, was about 60 min.

#### Materials and procedure

Participants memorised five memory items that appeared on screen simultaneously, at five distinct spatial locations. Depending on the experimental condition, participants may have been asked to prioritise one of these items in working memory. At the end of the trial, participants were probed to type the word that had appeared at one of the spatial locations during encoding. Participants were randomly assigned to either the Cue or Reward group. Each group was alternated at every session, which consisted of a maximum of six participants, such that all participants tested together pertained to the same experimental group.^3^

We administered the task using E-Prime 3 (Psychology Software Tools). The stimuli were simple four-letter French words, e.g., *pain* (in English, *bread*), presented in Courier New font, size 18, and all capital letters. We selected 64 words from the French-word database, *Lexique* (New et al., 2004), and only used single-syllable words which had no special characters (e.g., *é, à, ç*). Word frequency ranged between 2.57 and 304.3, with a mean of 38.79 (per million occurrences).^4^

**Figure 1.**
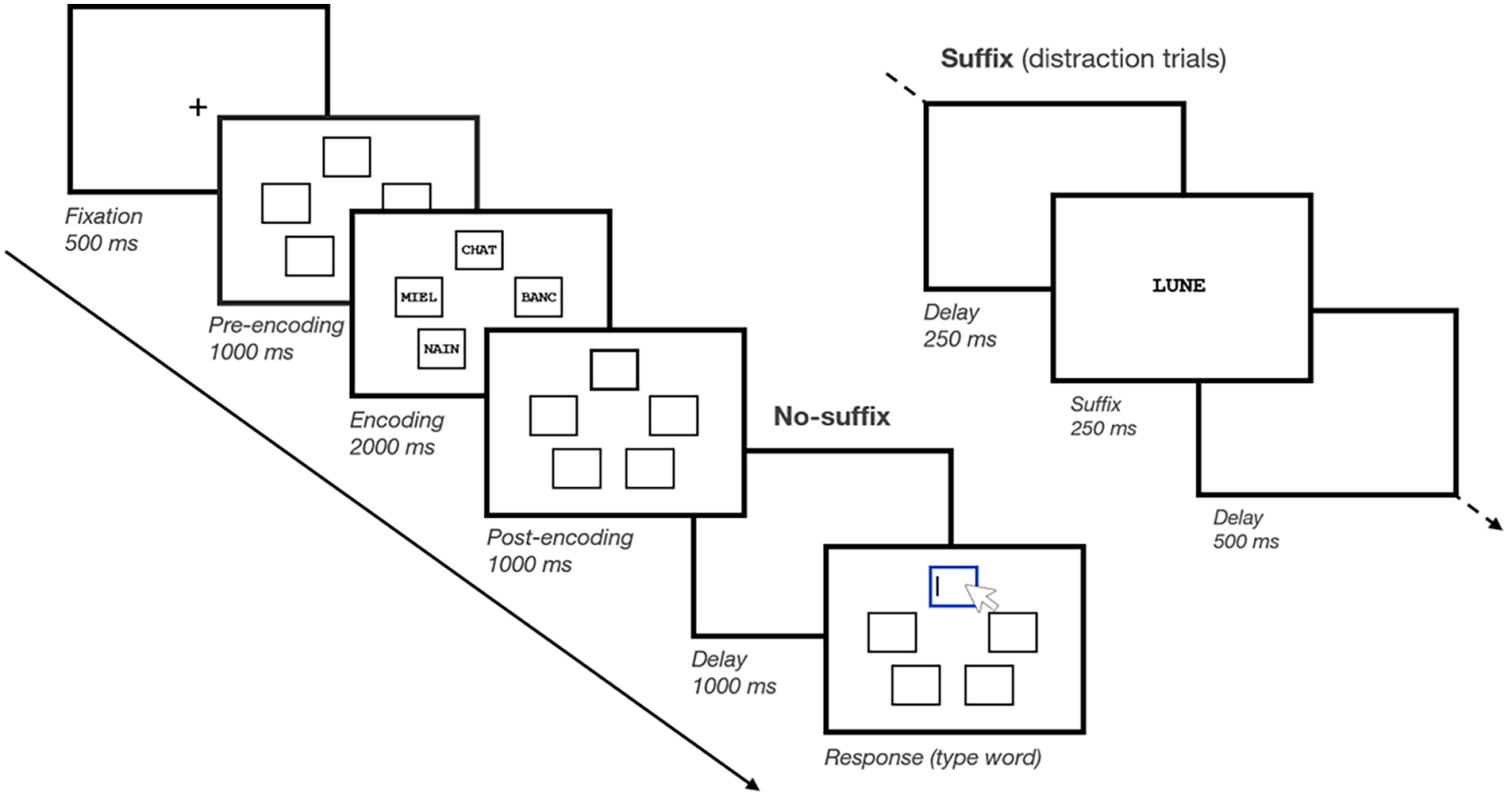
Schematic illustration of a trial in Experiment 1. Participants memorised five short words, shown in five different frames. These five frames were also shown empty, both before and after memory item presentation (i.e., pre-encoding and post-encoding). On No-prioritisation trials, none of the frames was bolded. On Pre-prioritisation trials, one of the five frames was bolded before item presentation. On Post-prioritisation trials, one of the five frames was bolded after item presentation (as illustrated here). On all trials, after a brief retention interval, memory was tested through cued recall. During the brief retention interval, either the screen remained blank or a suffix item was shown centrally on screen.

The task is shown in Figure 1. At the beginning of each trial, a fixation cross was presented for 500 ms. Next, a screen displayed five frames on an invisible pentagon, such that each frame appeared at five distinct locations, for 1000 ms. Then, five memory items appeared simultaneously inside each frame for 2000 ms (i.e., 400 ms encoding time per item), after which only the frames remained on screen for 1000 ms. In the *Pre-prioritisation* block, the prioritisation signal appeared prior to encoding, where one of the frames was highlighted by appearing with a thick black border. In the *Post-prioritisation* block, the prioritisation signal appeared following encoding, where one of the frames was highlighted by appearing with a thick black border. Finally, in the *No-prioritisation* block, no prioritisation signal appeared, and thus, none of the memory items were to be prioritised.

For both *Pre-prioritisation* and *Post-prioritisation* blocks, the spatial location of the thick-bordered frame was randomly determined on every trial, such that each memory item had an equal probability of being prioritised, i.e., 20%. However, the exact meaning of the prioritisation signal was manipulated between both Prioritisation mode groups. Participants in the Cue group were told that the bolded frame cued the spatial location of the to-be-tested item. Participants in the Reward group were told that the bolded frame signalled which item would result in winning more points if it was subsequently correctly recalled at test.

Prior to the probe screen, there was a brief delay of 1000 ms. In half of the trials, we included distraction, i.e., a plausible suffix (i.e., a word randomly drawn from the same set as the memory items but not included in the memory set of the current trial; see Allen & Ueno, 2018). For trials with distraction (i.e., Suffix trials), a 250-ms empty delay was presented, followed by the suffix word displayed for 250 ms at the centre of the screen, and finally, a 500-ms empty delay. For trials without distraction (i.e., No-suffix trials), the delay screen simply remained empty during the entire 1000 ms. At the end of the trial, the test screen appeared, where one of the frames was highlighted with a thick blue border. The mouse cursor also appeared on screen, prompting participants to type the word they had memorised at the probed spatial location. They recalled the probed word by typing it into the keyboard; their response was echoed on screen. Participants then pressed *Enter* to confirm their response. Even if they were not confident of their response, participants were encouraged to provide their best guess and to respond as quickly as possible. They were also allowed to use the *Backspace* key to correct their response.

In the Cue group, participants were told that the black bolded frame shown before or after encoding indicated which item was going to be tested at the end of the trial (100% valid cue). In the Reward group, participants were told that the black bolded frame shown before or after encoding indicated the spatial position of the memory item that was going to earn them a higher number of points if correctly recalled at the end of the trial. In the Reward group, following Allen and Ueno (2018), the prioritised item was assigned a point reward equal to the set size number. Therefore, correctly recalling the prioritised item resulted in earning 5 points, whereas correctly recalling any other item resulted in 1 point. Importantly, unlike the Cue group, the prioritisation signal in the Reward group was not predictive of which item would be subsequently tested (see Allen & Ueno, 2018; Hu et al., 2014; Vergauwe et al., 2023). Thus, for the Cue group, memory was always tested for the cued item (100% probability) on Pre-prioritisation and Post-prioritisation trials, whereas on No-prioritisation trials, all items were equally likely to be tested (20% probability). For the Reward group, however, all five items were equally likely to be tested (20% probability) on Pre-prioritisation, Post-prioritisation, and No-prioritisation trials. The meaning of the prioritisation signal was explained explicitly to the participants in the instructions prior to beginning the task. Finally, participants in the Reward group were also aware that the points represented entirely notional rewards.

At the beginning of the experiment, the main task was explained step by step. First, participants performed two practice trials without distraction. In the next two trials, the suffix item was added. Then four test trials followed, two with distraction and two without distraction, randomly intermixed. Before beginning each block (*Pre-prioritisation, Post-prioritisation*, or *No-prioritisation*), the exact conditions of that block were explained. Participants completed two practice trials before each block.

### Results

**Figure 2.**
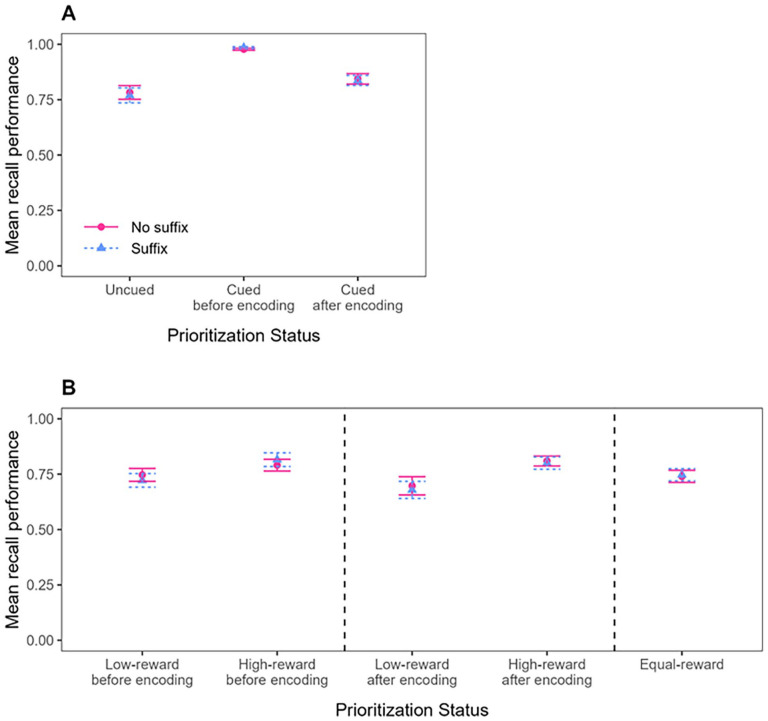
Mean recall performance in Experiment 1, as a function of Prioritisation mode (Cue-based prioritisation or Reward-based prioritisation), Distraction (No suffix vs. Suffix), and Prioritisation Status. For Cue-based prioritisation (Panel A), Prioritisation Status includes Uncued or Cued, with the Cued condition further distinguished by whether prioritisation occurred before or after encoding. For Reward-based prioritisation (Panel B), Prioritisation Status includes Low-reward, High-reward, or Equal-reward, with Low-reward and High-reward conditions further distinguished by whether prioritisation occurred before or after encoding. Error bars represent standard error of the mean.

Mean recall performance (proportion of correct responses to the test probes) for the Cue group and the Reward group is shown in Figure 2. In line with our preregistration, the data were analysed in five successive steps. All analyses were done using R (R Core team, 2021).

First, to examine whether prioritising information in verbal working memory had an impact on memory performance and on the distractor susceptibility of the information in question when using cue-based prioritisation, we analysed the data of the Cue group using the comparison that is typically examined in the Cue paradigm: the performance for prioritised items corresponded to performance observed for cued items and was extracted from trials on which a cue was presented (Pre-prioritisation and Post-prioritisation trials), whereas performance for unprioritized items corresponded to performance observed for uncued items and was extracted from trials on which no cue was presented (No-prioritisation trials; see Panel A in Figure 2). This analysis is referred to as “Cue group—conventional comparison.”^5^

Second, to examine whether prioritising information in verbal working memory had an impact on memory performance and on the distractor susceptibility of the information in question when using reward-based prioritisation, we analysed the data of the Reward group using the comparison that was most often used in the first studies using the Reward paradigm and examining the impact of reward on distractor susceptibility (e.g., Allen & Ueno, 2018; Hitch et al., 2018; Hu et al., 2014, 2016). Performance for prioritised items corresponded to performance observed for high-rewarded items and was extracted from trials on which a reward pattern was presented (Pre-prioritisation and Post-prioritisation trials), and in the same way, performance for unprioritized items corresponded to performance observed for low-rewarded items and was extracted from trials on which a reward pattern was presented (Pre-prioritisation and Post-prioritisation trials). This analysis is referred to as “Reward group—conventional comparison”^6^ because it is the approach that has been used in the earlier studies using the Reward paradigm.

In a third step, the impact of reward-based prioritisation on memory performance and on distractor susceptibility in verbal working memory was tested using an alternative comparison in which we analysed the data of the Reward group using a comparison that is more similar to the one typically examined in the Cue paradigm (and more similar to the comparison used in more recent studies using the Reward paradigm; e.g., Allen & Atkinson, 2021; Atkinson et al., 2018, 2019, 2021, 2022; Hu et al., 2023; Johnson & Allen, 2023; Roe et al., 2024). In this analysis, performance for prioritised items still corresponded to the performance observed for high-rewarded items and was still extracted from trials on which a reward pattern was presented (Pre-prioritisation and Post-prioritisation trials), but performance for unprioritized items corresponded now to performance observed for equally-rewarded items and was extracted from trials on which no reward pattern was presented (No-prioritisation trials). This analysis is referred to as “Reward group—alternative comparison” because it differs from the conventional approach described above.

In a fourth step, we aimed to compare the impact of prioritisation on distractor susceptibility between prioritisation modes. Therefore, we calculated vulnerability scores for prioritized and unprioritized items, for both cue-based and reward-based prioritisation (with data extracted from trials using the conventional comparisons), and compared these directly. Finally, in a last step, we tested more directly whether the observed pattern in verbal working memory corresponded to what was previously observed in visuospatial working memory (i.e., protected prioritised information in cue-based paradigms vs. particularly-vulnerable prioritised information in reward-based paradigms). To do so, an alternation index was calculated for both groups, which captured the change in vulnerability scores when prioritized and unprioritized items were compared. This last set of analyses is referred to as “Vulnerability scores” (see Vergauwe et al., 2023, for a similar analysis approach).

#### Cue group—conventional comparison

Mean recall performance in the Cue group was analysed by performing a repeated-measures Bayesian Analysis of Variance (BANOVA) with Prioritisation Timepoint (Pre-prioritisation, Post-prioritisation, or No-prioritisation) and Distraction (Suffix vs. No suffix) as within-subjects variables. The best model of the data in the Cue group was the Prioritisation Timepoint-only model (BF of 5.71 × 10^21^ against the null). Against our expectations, there was evidence against the main effect of Distraction (BF_01_ = 5.87), and the best model was almost 51 times better than the full model, which included the interaction of interest. As can be seen in Figure 2, Panel A, cueing improved memory performance, but presenting a suffix did not disrupt memory performance, and this pattern did not vary as a function of when the cue was presented. This was confirmed in additional analyses that examined the impact of cue-based prioritisation for the different timepoints separately (see Supplementary Materials 2).

#### Reward group—conventional comparison

Mean recall performance for the conventional comparison in the Reward group was analysed by performing a repeated-measures BANOVA with Prioritisation Timepoint (Pre-prioritisation vs. Post-prioritisation), Prioritisation Status (High-reward vs. Low-reward), and Distraction (Suffix vs. No suffix) as within-subjects variables. The best model of the data in the Reward group was the Prioritisation Status-only model (BF of 2.74 × 10^9^ against the null). Like in the Cue group, there was evidence against the main effect of Distraction (BF_01_ = 6.29), and the best model was almost 18 times better than the model that also included the main effect of Distraction and its interaction with Prioritisation Status. As can be seen in Figure 2, Panel B, rewarding an item improved its memory performance, relative to the items that were rewarded with only 1 point on these same trials, but presenting a suffix did not disrupt memory performance. This pattern did not vary as a function of when the reward pattern was presented. This was confirmed in additional analyses that examined the impact of reward-based prioritisation for the different timepoints separately (see Supplementary Materials 2).

#### Reward group—alternative comparison

In this comparison, unprioritized items were extracted from trials without a reward manipulation, in the same way as unprioritized items in the Cue paradigm are typically extracted from trials without a cue. Doing so, mean recall performance was now analysed by performing a repeated-measures BANOVA with Prioritisation Timepoint (Pre-prioritisation, Post-prioritisation, or No-prioritisation) and Distraction (Suffix vs. No suffix) as within-subjects variables, like in the Cue group. Like in the Cue group, the best model of the data was the Prioritisation Timepoint-only model (BF of 59.07 against the null). There was again evidence against the main effect of Distraction (BF_01_ = 5.56), and the best model was about 39 times better than the full model, which included the interaction of interest. As can be seen in Figure 2, Panel B, and similar to what we observed using the conventional comparison in the Reward group, rewarding improved memory performance, but presenting a suffix did not disrupt memory performance, and this pattern did not vary as a function of when the reward was presented. This was again confirmed in additional analyses that examined the data for the different timepoints separately (see Supplementary Materials 2).

#### Vulnerability scores

**Figure 3.**
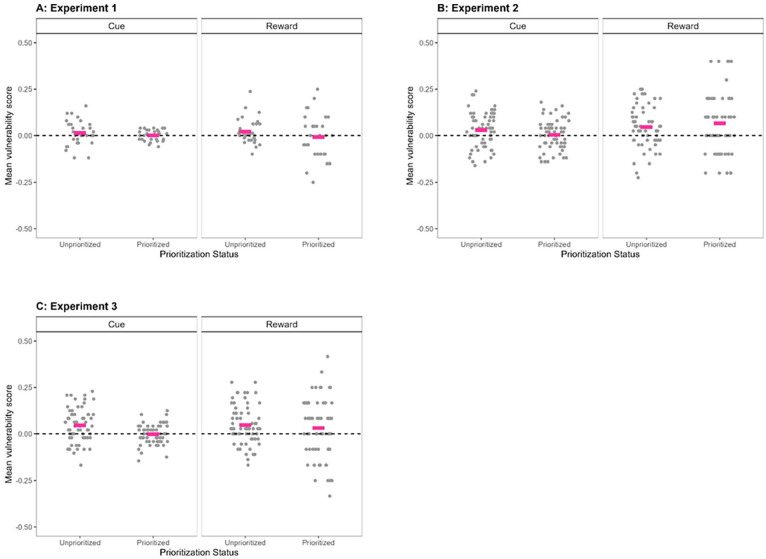
Mean vulnerability scores in Experiments 1–3 (mean recall performance in No-suffix condition minus mean recall performance in Suffix condition), as a function of Prioritisation mode (Cue-based prioritisation on the left, Reward-based prioritisation on the right), and Prioritisation Status (Unprioritized vs. Prioritised, corresponding to Uncued vs. Cued items, respectively, in the Cue group, and corresponding to Low-reward vs. High-reward items, respectively, in the Reward group). Grey points are showing individual data, and pink lines represent the means per condition. The dotted line represents a vulnerability score of 0; scores above this line indicate the presence of a disruptive effect of distraction on memory performance, whereas scores on or below this line indicate the absence of a disruptive effect of distraction on memory performance.

Until now, the distractor susceptibility results of the Cue and Reward groups were analysed separately. In this last set of analyses, we aimed to compare them more directly. To do so, we first calculated, per participant in each group, a vulnerability score for prioritised and unprioritized items separately (i.e., memory performance in the No-suffix condition minus memory performance in the Suffix condition). As preregistered, we used the aforementioned conventional comparisons for these calculations (see Supplementary Materials 2 for more details). These vulnerability scores are shown in Figure 3, Panel A, and were analysed by performing a BANOVA with Prioritisation Status (Prioritised vs. Unprioritized) as a within-subjects variable and Prioritisation Mode (Cue vs. Reward) as a between-subjects variable. The best model of the data was found to be the null model (and this model was 21.63 times better than the full model including the interaction of interest). This implies that the vulnerability scores should be considered as invariant across Prioritisation Status and Prioritisation Mode.

For the last preregistered analysis, we calculated an alteration index, which captures the change in vulnerability between prioritized and unprioritized information by subtracting vulnerability scores for unprioritized items from those for prioritised items, separately for each participant and each group. When this alteration index is 0, it indicates that prioritised and unprioritized items are just as vulnerable to distraction (i.e., equal vulnerability scores for prioritised and unprioritized items). When this alteration index is above 0, it indicates that prioritised items are more vulnerable than unprioritized items (i.e., higher vulnerability scores for prioritised items than for unprioritized items). Finally, when the alteration index is below 0, it indicates that prioritised items are protected from interference, relative to unprioritized items (i.e., lower vulnerability scores for unprioritized items than for prioritised items).

Using three one-sided *t*-tests, we showed that (1) the alteration index was not smaller than 0 in the Cue group (BF_01_ = 2.03; i.e., weak evidence against a protected state for prioritised information), (2) the alteration index was not larger than 0 in the Reward group (BF_01_ = 10.67; i.e., strong evidence against a particularly vulnerable state for prioritised information), and (3) the reward manipulation did not result in a larger alteration index than the cue manipulation (BF_01_ = 5.61; i.e., modest evidence that the way in which information is prioritised does not influence the vulnerability of the prioritised information in the same direction as observed in visuospatial working memory). This was confirmed in additional analyses (see Supplementary Materials 2).

### Discussion

In Experiment 1, we observed clear memory boosts for information that was prioritised in verbal working memory. Improved memory performance was evident with both cue-based and reward-based prioritisation, and it occurred regardless of whether the priority signal was presented before or after encoding. For reward-based prioritisation, the memory boost was observed both when high-reward items were compared to low-reward items (as often done in earlier reward-based studies) and when high-reward items were compared to equal-reward items (as often done in more recent reward-based studies, as well as in most cue-based studies). The data were less conclusive, however, when it comes to the effects of prioritisation on distractor susceptibility. In particular, perceptual interference did not affect memory performance, preventing us from investigating whether the negative impact of perceptual interference is affected by the priority status of the memorised information and whether this varies between cue-based and reward-based prioritisation. Following up on this experiment, we planned two additional experiments that aimed to assess memory performance and distractor susceptibility, with modifications to increase the likelihood of detecting any detrimental effects of perceptual interference. In Experiment 2, the modifications concerned the nature of the perceptual interference; in Experiment 3, the modifications primarily targeted the nature of the memory task.

## Experiment 2

Experiment 2 made the perceptual interference harder to ignore by (1) presenting the interfering item both visually and auditorily, (2) doubling its presentation time, and (3) presenting it at all locations where a memory item had previously been presented, instead of a single, centrally presented interfering item. We again compared cue-based vs. reward-based prioritisation but no longer included a manipulation of when prioritisation was induced—it was always induced after encoding because this timepoint allowed us to zoom in specifically on the effects of prioritisation occurring during retention (i.e., when information has been encoded and can be assumed to be represented in working memory).

### Method

The preregistration for Experiment 2 can be found at https://osf.io/r7bsd.

#### Participants and design

Our sample size was determined by the Bayesian sequential hypothesis testing procedure described in Supplementary Materials 1. A total of 125 undergraduate students (108 female, 17 male, 0 other; mean age = 22 years) were recruited from the University of Geneva and participated in exchange for partial course credit. All participants had normal or corrected-to-normal vision. No performance-based exclusions were applied, but one participant was excluded due to a technical error that occurred during the experiment. Thus, our final sample contains the data of 124 participants, including 62 participants per group.

We implemented a 2 × 2 × 2 design, with Prioritisation Mode (Cue vs. Reward) as the between-subjects variable and Prioritisation Presence (Post-prioritisation vs. No-prioritisation) and Distraction (Suffix vs. No suffix) as within-subjects variables. Note that in comparison to Experiment 1, we no longer included the *Prioritisation Timepoint* manipulation—when a prioritisation signal was presented, it was always presented post-encoding. In each group (Cue and Reward), Prioritisation Presence was implemented in blocks, and the order of the blocks was counterbalanced. In total, participants completed 200 experimental trials. As in Experiment 1, these trials were presented in blocks of 100 trials. Within each block (*Post-prioritisation* and *No-prioritisation*), *Suffix* and *No-suffix* trials were equally likely to occur (50 each) and were randomly intermixed. Total experiment runtime, including consent and on-screen instructions, was about 35 and 40 min for the Cue and Reward groups, respectively.

#### Materials and procedure

**Figure 4.**
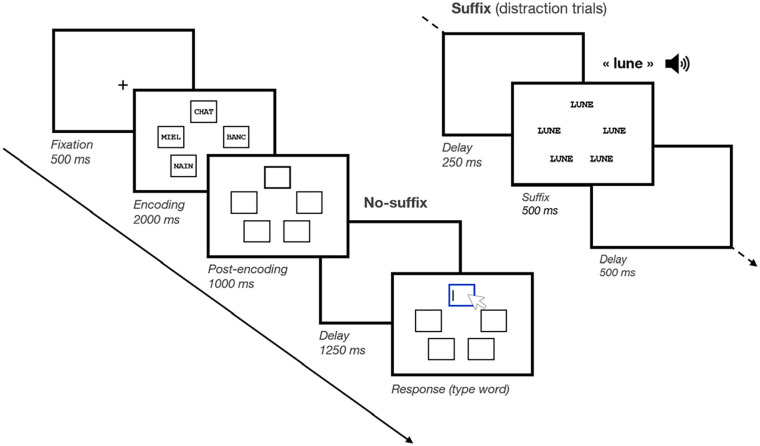
Schematic illustration of a trial in Experiment 2. Participants memorised five short words, shown in five different frames. These five frames were also shown empty after memory item presentation (i.e., post-encoding). On No-prioritisation trials, none of the frames was bolded; on Post-prioritisation trials, one of the five frames was bolded after item presentation (as illustrated here). On all trials, after a brief retention interval, memory was tested through cued recall. During the brief retention interval, either the screen remained blank or a suffix item was shown in all five locations.

We administered the task using E-Prime 3 (Psychology Software Tools) and used the same stimuli as Experiment 1. The main task was the same as that in Experiment 1, except for the following modifications (see Figure 4). First, since we no longer included the *Pre-prioritisation* condition, we removed the pre-encoding delay in between the fixation cross and memory item presentation. Second, we implemented three changes to make the distraction more difficult for participants to ignore. Specifically, suffix items were presented (1) not only visually but also auditorily through an audio recording, (2) for 500 ms rather than 250 ms, and (3) at all five spatial locations on screen rather than only centrally. Thus, on Suffix trials, a 250-ms empty delay was presented after encoding, followed by the same suffix word presented for 500 ms at all five locations on screen while being echoed auditorily,^7^ and finally, a final 500-ms empty delay was presented prior to memory test. On No-suffix trials, the delay screen simply remained empty during 1250 ms.

For the Post-prioritisation block, the spatial location of the black bolded frame was determined randomly on every trial, such that each memory item had equal probability of being prioritised (20%). Like in Experiment 1, in the Cue group, the prioritised item was always tested (100% valid cue), and in the Reward group, the single prioritised item was worth 5 points when correctly recalled (all other items were still worth 1 point) but had the same probability of being tested as the other items (20%).

### Results

**Figure 5.**
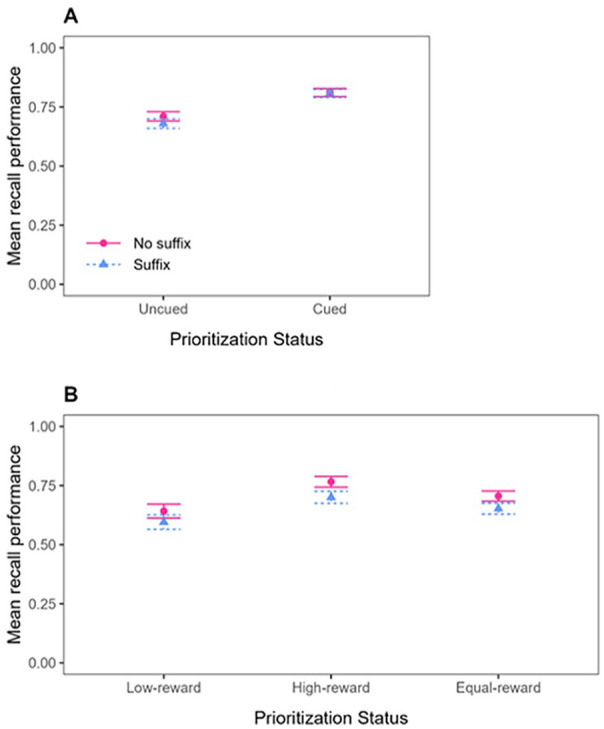
Mean recall performance in Experiment 2, as a function of Prioritisation mode (Cue-based prioritisation or Reward-based prioritisation), Distraction (No suffix vs. Suffix), and Prioritisation Status. For Cue-based prioritisation (Panel A), Prioritisation Status includes Uncued or Cued. For Reward-based prioritisation (Panel B), Prioritisation Status includes Low-reward, High-reward, or Equal-reward. Error bars represent standard error of the mean.

Mean recall performance (proportion of correct responses to the test probes) for the Cue group and the Reward group is shown in Figure 5. In line with our preregistration, the data were analysed in five successive steps. These five steps correspond to the ones described for Experiment 1, except that now, there were only Post-prioritisation and No-prioritisation trials (when a priority signal was presented, it was always after encoding).

#### Cue group—conventional comparison

Mean recall performance in the Cue group was analysed by performing a repeated-measures BANOVA with Prioritisation Presence (Post-prioritisation vs. No-prioritisation) and Distraction (Suffix vs. No suffix) as within-subjects variables. The best model of the data in the Cue group was the Prioritisation Presence-only model (BF of 1.92 × 10^24^ against the null). The evidence regarding the main effect of Distraction remained inconclusive (BF_01_ = 1.20), and the best model was about two times better than the full model which included the interaction of interest. Thus, even though Figure 5, Panel A, descriptively shows a pattern whereby the uncued items (from No-prioritisation trials) are disrupted by the suffix, whereas the cued item (from Post-prioritisation trials) are not, the BANOVA did not reveal evidence for the interaction of interest. However, un-preregistered, one-sided *t*-tests of vulnerability scores in the Cue group did reveal moderate evidence for a negative impact of interference on memory performance on uncued items (BF_10_ = 5.49) and against a negative impact of interference on memory performance on cued items (BF_01_ = 5.33), providing at least some evidence for the expected pattern in the Cue group.

#### Reward group—conventional comparison

Mean recall performance for the conventional comparison in the Reward group was analysed by performing a repeated-measures BANOVA with Prioritisation Status (High-reward vs. Low-reward, both from Post-prioritisation trials) and Distraction (Suffix vs. No suffix) as within-subjects variables. The best model of the data in the Reward group was the model with the two main effects (without the interaction). There was strong evidence for including the Prioritisation Status variable (BF_10_ = 1.30 × 10^8^) as well as for including the main effect of Distraction (BF_10_ = 30.53). The evidence against the interaction of interest was modest (BF_01_ = 4.44).

As can be seen in Figure 5, Panel B, rewarding an item improved its memory performance, relative to the items that were rewarded with only 1 point on these same trials, and memory performance of both high-reward and low-reward items appeared disrupted by the suffix. Un-preregistered, one-sided *t*-tests of vulnerability scores in the Reward group revealed strong evidence for a negative impact of interference on memory performance of both low-reward and high-reward items, BF_10_ = 35.32 and BF_10_ = 26.06, respectively. It appears that high-reward and low-reward items were both vulnerable to the suffix and to the same extent.

#### Reward group—alternative comparison

Mean recall performance for the alternative comparison in the Reward group was analysed by performing a repeated-measures BANOVA with Prioritisation Presence (Post-prioritisation vs. No-prioritisation) and Distraction (Suffix vs. No suffix) as within-subjects variables, like in the Cue group. Like in the conventional comparison of the Reward group, but unlike the Cue group, the best model of the data was the model with the two main effects (without the interaction). There was strong evidence for including the Prioritisation Status variable (BF_10_ = 412) as well as for including the main effect of Distraction (BF_10_ = 2087), and there was modest evidence against the interaction of interest (BF_01_ = 4.80). As can be seen in Figure 5, Panel B, and similar to what we observed using the conventional comparison in the Reward group, high-reward and equal-reward items both appeared vulnerable to the suffix and to the same extent.

#### Vulnerability scores

We calculated a vulnerability score for prioritised and unprioritized items separately, as described in Experiment 1, using the aforementioned conventional comparisons for these calculations. These vulnerability scores are shown in Figure 3, Panel B, and were analysed by performing a BANOVA, with Prioritisation Status (Prioritised vs. Unprioritized) as a within-subjects variable and Prioritisation Mode (Cue vs. Reward) as a between-subjects variable. The best model was the Prioritisation Mode-only model (BF of 2.55 against the null). There was quite some evidence against a main effect of Prioritisation Status (BF_01_ = 7.3), and the best model was about 10 times better than the full model including the interaction of interest.

For the last preregistered analysis, we calculated the alteration index as described in Experiment 1. Using three one-sided *t*-tests, we showed that (1) the data were inconclusive as to whether the alteration index was smaller than 0 in the Cue group (BF_10_ = 1.15; i.e., inconclusive evidence for a protected state for prioritised information), (2) the alteration index was not larger than 0 in the Reward group (BF_01_ = 3.47; i.e., modest evidence against a particularly vulnerable state for prioritised information), and (3) the data were inconclusive as to whether the reward manipulation resulted in a larger alteration index than the cue manipulation (BF_10_ = 1.03; i.e., inconclusive evidence for prioritisation mode influencing the vulnerability of prioritised information in the same direction as observed in visuospatial working memory).

A set of un-preregistered, one-sided *t*-tests, one for each vulnerability score shown in Figure 3, Panel B, tested whether there was evidence in the data for the score being larger than 0, i.e., for the information in that particular state being vulnerable. This revealed evidence for a negative impact of interference on memory performance for all types of items except for cued items. Indeed, we observed BFs of 5.49, 35.32, and 26.06, for a negative impact of the suffix for uncued items, low-reward items, and high-reward items, respectively, but a BF of 5.33 against a negative impact of the suffix for cued items.

### Discussion

Like in Experiment 1, we observed a memory boost for information prioritised within verbal working memory. In particular, we observed a clear memory boost for both cue-based and reward-based retrospective prioritisation. The boost for high-reward items was evident when compared to low-reward items (as often done in early reward-based studies), as well as when compared to equal-reward items (as typically done in cue-based studies and in more recent reward-based studies). Relative to Experiment 1, we made several modifications to the perceptual interference to make it harder for participants to ignore the distracting information during retention in Experiment 2. Despite these modifications, the evidence for a detrimental effect of the distraction was still not convincing in our preregistered analyses in the Cue group. Only in un-preregistered follow-up analyses, there was some evidence (1) for a negative impact of perceptual interference on uncued items and (2) against such a negative impact on cued items. Taken together, this represents some evidence for reduced distractor susceptibility for prioritised information with cue-based prioritisation of verbal memory items, but the evidence should be considered as weak.

In the Reward group, we *did* find convincing evidence for a negative impact of perceptual interference on memory performance in our preregistered analysis. However, this effect of distraction was not different between prioritised and unprioritized information, both when high-reward items were compared to low-reward items and when high-reward items were compared to equal-reward items (preregistered and un-preregistered analyses). Thus, distractor susceptibility of verbal information does not appear affected by reward-based prioritisation. Overall, even though Experiment 2 provided more evidence for a negative impact of perceptual interference on memory performance, there was only limited evidence for reduced distractor susceptibility of prioritised information.

## Experiment 3

Like Experiment 2, Experiment 3 directly followed up on the lack of an effect of perceptual interference on memory performance in Experiment 1. Whereas in Experiment 2, we did so by manipulating the nature of the perceptual interference, in Experiment 3, we targeted primarily the nature of the memory task. In Experiments 1 and 2, black words were shown in different locations, and location probes were used at test, instructing participants to type the word that had been presented at the probed location. In several visuospatial studies, however, and especially in studies that used reward-based prioritisation in visuospatial working memory, coloured shapes were to be memorised, and either colour or shape probed recall of the other feature at test (e.g., Allen & Ueno, 2018; Hu et al., 2014, 2016; Vergauwe et al., 2023). There are several differences between those studies and our verbal analogue in Experiment 1, other than the visuospatial vs. verbal nature of the memoranda, that could potentially play a role in the lack of a negative effect of perceptual interference in Experiment 1.

For example, in Experiment 1, only word identity needed to be remembered, whereas studies with coloured shapes required remembering two features (colour and shape) and their binding. Ueno et al. (2011) showed that irrelevant distractors have a greater negative impact on objects comprised of bound features than on individual features. Moreover, Experiment 1 used location to probe recall, whereas other studies used colour or shape to probe recall (e.g., Allen & Ueno, 2018; Vergauwe et al., 2023). Since location is considered a special feature in working memory (e.g., Schneegans et al., 2023; Treisman & Zhang, 2006), we thought it is possible that the use of location to probe recall may have affected our observations in Experiment 1. Overall, it was not our goal to pinpoint the exact reason for not finding a detrimental effect of distraction in Experiment 1, but rather to modify our verbal experiment to make it more similar to the studies in visuospatial working memory that *did* find negative effects of perceptual interference and were hence able to test whether distractor susceptibility varies between information. To mimic those memory tasks more closely in the verbal domain, Experiment 3 presented coloured words at encoding (instead of black words), and a colour blob (instead of a location) probed recall of the word at test (e.g., type “CAT” when a green blob is displayed at test, and “CAT” had been shown in green at encoding). We again compared cue-based vs. reward-based prioritisation, and like in Experiment 2, the prioritisation signal was always presented after encoding.

### Method

The preregistration for Experiment 3 can be found at https://osf.io/2k6qy.

#### Participants and design

Our sample size was determined by the Bayesian sequential hypothesis testing procedure described in Supplementary Materials 1. A total of 144 undergraduate students (118 female, 26 male, 0 other; mean age = 23 years) were recruited from the University of Geneva and participated in exchange for partial course credit. All participants had normal or corrected-to-normal vision. No performance-based exclusions were applied, but 11 participants were excluded due to either a fire drill evacuation during testing (six) or technical errors (five). Thus, our final sample contains the data of 133 participants, including 69 and 64 participants in the Cue and Reward groups, respectively.

We implemented a 2 × 2 × 2 design, with Prioritisation Mode (Cue vs. Reward) as between-subjects variable and Prioritisation Presence (Post-Prioritisation vs. No-prioritisation) and Distraction (Suffix vs. No suffix) as within-subjects variables. In both groups (Cue and Reward), Prioritisation Presence was implemented in blocks, and the order of the blocks was counterbalanced across participants. In total, participants completed 192 experimental trials. As in Experiments 1 and 2, these trials were presented in blocks; this time of 96 trials each. Within each block, *Suffix* and *No-suffix* trials were equally likely (48 each), and they were randomly intermixed. Total experiment runtime, including consent and on-screen instructions, was about 35 and 40 min for the Cue and Reward groups, respectively.

#### Materials and procedure

**Figure 6.**
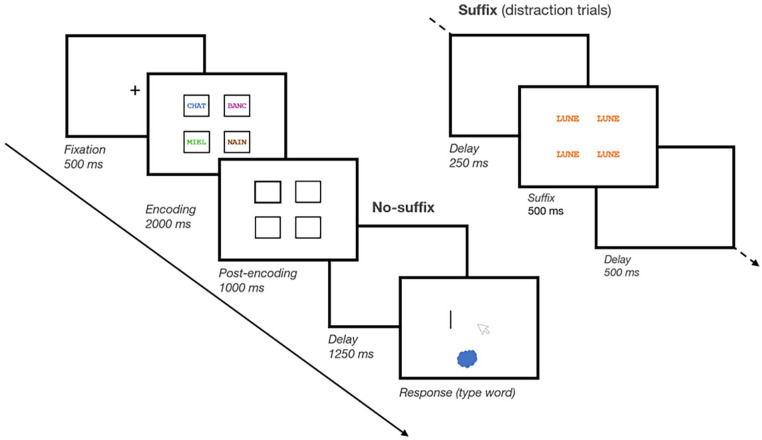
Schematic illustration of a trial in Experiment 3. Participants memorised four short words, shown in four different colours and in four different frames. These four frames were also shown empty after memory item presentation (i.e., post-encoding). On No-prioritisation trials, none of the frames was bolded, and on Post-prioritisation trials, one of the four frames was bolded after item presentation (as illustrated here). On all trials, after a brief retention interval, memory was tested through cued recall. During the brief retention interval, either the screen remained blank or a suffix item was shown in all four locations.

We administered the task using E-Prime 3 (Psychology Software Tools). Whereas to-be-memorised words were presented in black in Experiments 1 and 2, they were presented in different colours in Experiment 3 (see Figure 6). In particular, we created a pool of 64 word-colour stimulus pairs, based on pairing eight colours and eight words, while avoiding salient stimulus pairs (i.e., pairing BLOOD with red would be considered a salient pair). We selected eight words from the pool used in Experiments 1 and 2 (*ange, arme, coin, cour, gare, lait, main*, and *trou*, corresponding to the French words for *angel, weapon, corner, courtyard, train station, milk, hand*, and *hole*, respectively), and the eight colours were those used in Experiment 2 (red, blue, yellow, green, aqua, purple, grey, and brown) of Vergauwe et al. (2023).

The paradigm was most similar to that of Experiment 2, except for the following differences related to set size, distraction, and memory test. First, four memory items were shown on each trial (instead of five). These items were displayed simultaneously, for 2000 ms (i.e., 500 ms per item), at four distinct spatial locations on an invisible square, and each item was again enclosed by a frame. For the *Post-prioritisation* block, the spatial location of the black bolded frame was again randomly determined on every trial, such that each memory item had an equal probability of being prioritised (this was now 25% instead of 20% because there were now four memory items instead of five). As in Experiments 1 and 2, the prioritised item was always tested in the Cue group. In the Reward group, as in the previous experiments, the prioritised item was assigned a point reward equal to the set size number, and thus, correctly recalling the prioritised item resulted in earning 4 points, whereas correctly recalling any of the other items resulted in earning 1 point (all four items had equal probability of being tested).

Second, concerning the distraction, we maintained suffix presentation time at 500 ms as in Experiment 2 but no longer presented it auditorily. Instead, we only displayed the suffix item visually and at all four spatial locations. In trials with distraction, a suffix item was presented as a coloured word. For each trial, a word was selected from the pool of eight words (excluding any words used as memory items in that trial). Similarly, the word’s colour was selected from the pool of eight colours (excluding any colours assigned to memory items in the same trial). Finally, for the memory test, we used an item’s colour rather than spatial location to probe participants’ memory. Thus, at the end of a trial, when the probe screen appeared, a coloured blob and mouse cursor appeared on screen, prompting participants to type the word they had memorised that had appeared in the probed colour at encoding.

### Results

**Figure 7.**
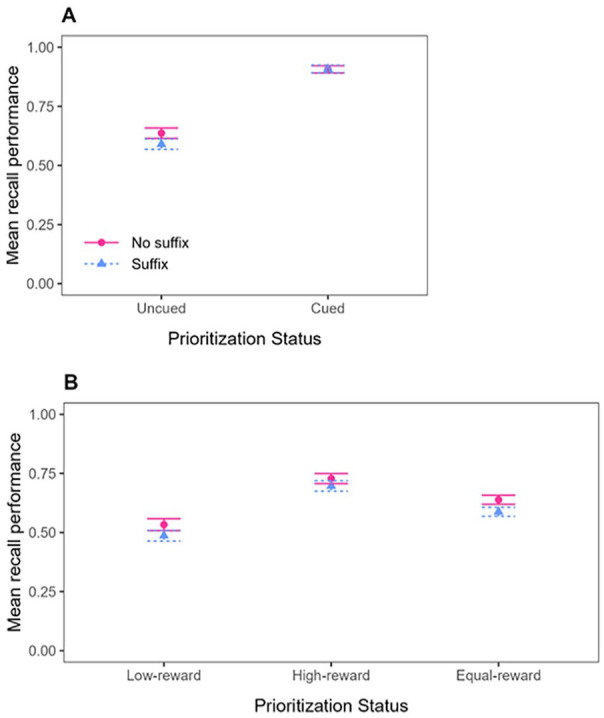
Mean recall performance in Experiment 3, as a function of Prioritisation mode (Cue-based prioritisation or Reward-based prioritisation), Distraction (No suffix vs. Suffix), and Prioritisation Status. For Cue-based prioritisation (Panel A), Prioritisation Status includes Uncued or Cued. For Reward-based prioritisation (Panel B), Prioritisation Status includes Low-reward, High-reward, or Equal-reward. Error bars represent standard error of the mean.

The mean recall performance (proportion of correct responses to the test probes) for the Cue and Reward groups is shown in Figure 7. In line with our preregistration, the data were analysed in five successive steps (described in detail in the results section of Experiment 2).

#### Cue group—conventional comparison

Mean recall performance in the Cue group was analysed by performing a repeated-measures BANOVA with Prioritisation Presence (Post-prioritisation vs. No-prioritisation) and Distraction (Suffix vs. No suffix) as within-subjects variables. The best model of the data in the Cue group was the Prioritisation Presence-only model (BF of 8.82 × 10^58^ against the null). As in Experiment 2, the evidence regarding the main effect of Distraction remained inconclusive (BF_01_ = 1.53), and the best model was only slightly better (1.28 times) than the full model which included the interaction of interest. Thus, even though Figure 7, Panel A, descriptively shows a pattern whereby the uncued items (from No-prioritisation trials) are disrupted by the suffix, whereas the cued item (from Post-prioritisation trials) is not, the BANOVA did not reveal evidence for the interaction of interest. However, like in Experiment 2, un-preregistered, one-sided *t*-tests of vulnerability scores in the Cue group (described below) did reveal very strong evidence for a negative impact of interference on memory performance on uncued items (BF_10_ = 1048) and strong evidence against a negative impact of interference on memory performance on cued items (BF_01_ = 9.73), providing some evidence for the expected pattern in the Cue group.

#### Reward group—conventional comparison

Mean recall performance for the conventional comparison in the Reward group was analysed by performing a repeated-measures BANOVA with Prioritisation Status (High-reward vs. Low-reward, both from Post-prioritisation trials) and Distraction (Suffix vs. No suffix) as within-subjects variables. The best model of the data in the Reward group was the model with only the main effect of Prioritisation Status. There was strong evidence for including the Prioritisation Status variable (BF_10_ = 2.90 × 10^17^). The data remained inconclusive regarding the main effect of Distraction (BF_01_ = 1.04), and there was moderate evidence against the interaction of interest (BF_01_ = 5.32). As can be seen in Figure 7, Panel B, both high-reward and low-reward items appear to be somewhat disrupted by the suffix. As described below, un-preregistered, one-sided *t*-tests of vulnerability scores in the Reward group revealed strong evidence for a negative impact of interference on memory performance of low-reward items, BF_10_ = 66.66, but remained inconclusive regarding a negative impact of interference on memory performance of high-reward items, BF_01_ = 1.27.

#### Reward group—alternative comparison

In addition, we performed a repeated-measures BANOVA with Prioritisation Presence (Post-prioritisation vs. No-prioritisation) and Distraction (Suffix vs. No suffix) as within-subjects variables, like in the Cue group. Unlike the conventional comparison of the Reward group and the Cue group, the best model of the data now included the main effect of Distraction; the best model was the model with the two main effects (without the interaction). There was strong evidence for including the Prioritisation Presence variable (BF_10_ = 7.66 × 10^6^), and there was moderate evidence for including the main effect of Distraction (BF_10_ = 3.51). There was also moderate evidence against the interaction of interest (BF_01_ = 4.39). As seen in Figure 7, Panel B, high-reward and equal-reward items both appeared vulnerable to the suffix and to the same extent.

#### Vulnerability scores

We calculated a vulnerability score for prioritised and unprioritized items separately, as described in Experiments 1 and 2. These vulnerability scores are shown in Figure 3, Panel C, and were analysed by performing a BANOVA with Prioritisation Status (Prioritised vs. Unprioritized) as a within-subjects variable and Prioritisation Mode (Cue vs. Reward) as a between-subjects variable. The best model was the Prioritisation Status-only model (BF of 3.63 against the null). There was modest evidence against a main effect of Prioritisation Mode (BF_01_ = 3.38), and the best model was about eight times better than the full model including the interaction of interest.

For the last preregistered analysis, we calculated the alteration index, as described in Experiments 1 and 2. Using three one-sided *t*-tests, we established that (1) there was very strong evidence for the alteration index being smaller than 0 in the Cue group (BF_10_ = 853; i.e., very strong evidence for a protected state for prioritised information); (2) there was strong evidence against the alteration index being larger than 0 in the Reward group (BF_01_ = 11.25; i.e., strong evidence against a particularly vulnerable state for prioritised information); and (3) the data remained inconclusive as to whether the reward manipulation resulted in a larger alteration index than the cue manipulation (BF_01_ = 1.49; i.e., inconclusive evidence as to whether prioritisation mode influenced the vulnerability of prioritised information in the same direction as observed in visuospatial working memory).

A set of un-preregistered, one-sided *t*-tests, one for each vulnerability score shown in Figure 3, Panel C, assessed the evidence in the data for the score being larger than 0, i.e., for the information in that particular state being vulnerable. As in Experiment 2, this revealed evidence for a negative impact of interference on memory performance for uncued and for low-reward items (BFs of 1048 and 66.66, respectively), together with evidence against a negative impact of the suffix for cued items (BF of 9.73). However, the data of Experiment 3 remained inconclusive regarding a negative impact of the suffix for high-reward items (BF_01_ = 1.27).

### Discussion

Like in Experiment 1 and 2, we found clear evidence for a memory boost with both cue-based and reward-based prioritisation, and again for both comparisons in the Reward group. Compared to Experiment 1, we made several changes, primarily concerned with the nature of the memory task. Like in Experiment 2, the evidence for a negative effect of perceptual interference was still inconclusive in the preregistered analyses in the Cue group. It was again only the un-preregistered follow-up analyses that revealed evidence (1) for a detrimental impact of distraction on memory performance for uncued and (2) against such an effect for cued items. Thus, there is some evidence for reduced distractor susceptibility for prioritised information with cue-based prioritisation in verbal working memory, but this evidence is not particularly strong.

Turning to the Reward group, the data remained inconclusive as to whether perceptual interference affects memory performance in the preregistered analysis of high-reward and low-reward items. Un-preregistered follow-up analyses showed evidence for a negative impact of distraction on memory performance for low-reward items but remained inconclusive as to the effect on memory performance for high-reward items. Although one could interpret this as reduced distractor susceptibility for prioritised information through reward-based prioritisation, we consider the evidence particularly weak (only in un-preregistered analyses, and no clear evidence *against* a negative impact for the prioritised information) and, thus, refrain from strong conclusions. When comparing high-reward to equal-reward items, we *did* find evidence for a detrimental effect of perceptual interference in our preregistered analysis, but there was also evidence for this effect being similar for high-reward vs. equal-reward items. Taken together, it appears that distractor susceptibility of verbal information was not substantially affected by reward-based prioritisation in Experiment 3. As such, the results of both Experiments 2 and 3 seem to indicate that distractor susceptibility of memorised information is not drastically influenced by whether the information was prioritised or not.

## Discussion Experiments 1–3

The findings from Experiments 1 to 3 indicate that memory for prioritised information in verbal working memory benefits consistently from both cue-based and reward-based retrospective prioritisation, as reflected in its enhanced memory performance. However, regarding the second anticipated benefit of directed attention during retention—reduced distractor susceptibility—our data from these experiments do not provide much evidence in support of this effect. In particular, across our experiments and experimental groups, perceptual interference did not impact memorised information as generally and as drastically as could be expected based on the literature. Moreover, when perceptual interference *was* found to affect memory performance, there was little or only weak evidence to suggest that distractor susceptibility was different between prioritized and unprioritized information in verbal working memory. In particular, only with cue-based prioritisation did we find some, rather weak, evidence suggesting that prioritised information is less affected by perceptual interference than unprioritized information. With reward-based prioritisation, our data indicate that prioritised and unprioritized information are equally affected by perceptual interference. Thus, while there is clear evidence for a memory boost for verbal information prioritised during retention, the evidence for reduced susceptibility of prioritised information is weak and limited to cue-based prioritisation.

In what follows, we focus more in-depth on the memory boost findings, which were robust across our experiments and prioritisation modes.^8^ The relevant mean values of memory performance are shown in Table 2 (only post-encoding prioritisation).

**Table 2.** Table reporting mean recall performance (percentage correct) for unprioritized and prioritised information in Experiments 1–5, along with the resulting boost (performance increase from unprioritized to prioritised) using the conventional comparison for cue-based (uncued vs. cued) and reward-based prioritisation (low-reward vs. high-reward) prioritisation, as well as the alternative comparison for reward-based prioritisation (equal-reward vs. high-reward).

		Cue-based prioritisation	Reward-based prioritisation
**E1**			
	Unprioritized information	78%	69% (low-reward) 74% (equal-reward)
	Prioritised information	84%	81%
	→ Boost	6%	12% (conventional) 7% (alternative)
**E2**			
	Unprioritized information	71%	64% (low-reward) 71% (equal-reward)
	Prioritised information	81%	77%
	→ Boost	10%	12% (conventional) 6% (alternative)
**E3**			
	Unprioritized information	64%	53% (low-reward) 64% (equal-reward)
	Prioritised information	91%	73%
	→ Boost	27%	20% (conventional) 9% (alternative)
**E4**			
	Unprioritized information	53%	48% (low-reward) 53% (equal-reward)
	Prioritised information	64%	57%
	→ Boost	12%	9% (conventional) 4% (alternative)
**E5**			
	Unprioritized information	73%	64% (low-reward) 70% (equal-reward)
	Prioritised information	78%	76%
	→ Boost	5%	12% (conventional) 7% (alternative)

A closer examination of the memory boosts in Experiments 1–3 suggests that the magnitude of these boosts is quite similar across cue-based and reward-based prioritisation, particularly when using the conventional comparison methods (averaging across Experiments 1–3, the increase in performance was 14 percentage points for cue-prioritisation and 15 percentage points for reward-based prioritisation). This similarity in the memory boosts associated with cue-based and reward-based prioritisation is different from what has been observed in visuospatial working memory, where the memory boost was often larger and more consistent for retrospective cue-based than for retrospective reward-based prioritisation when using the conventional comparisons (see Supplementary Materials 3 for details on the observations in visuospatial working memory). Based on this observation, we decided to focus exclusively on the memory boost in the last two experiments. Specifically, the goal of Experiments 4 and 5 was to understand whether the apparent difference between the verbal and visuospatial experiments reflects a domain-related difference, or whether it might be explained by differences in overall memory performance (Experiment 4) or response reconfiguration demands (Experiment 5) between the verbal and visuospatial experiments.

The reason for doing so is as follows. The overall goal of the current study was to examine whether the benefits of holding information in the focus of attention during retention extend from the visuospatial to the verbal domain of working memory. Thus, it was important to examine whether the same pattern of consequences across different prioritisation modes observed in visuospatial working memory also occurs in verbal working memory. If the memory boost for prioritised information is consistently larger for cue-based prioritisation than for reward-based prioritisation in both domains, it suggests the operation of a domain-general focus of attention in working memory, as well as an additional domain-general process that can account for the larger boost for cue-based prioritisation than for reward-based prioritisation in both domains. However, if the memory boost is similar for both prioritisation modes in verbal working memory, as suggested by Experiments –3, it indicates a domain-general focus of attention in working memory along with an extra process that specifically enhances cued visuospatial information.

## Experiment 4

In our verbal experiments, memory performance was considerably higher (roughly between 75% and 100%) than that for similar visuospatial experiments (roughly between 50% and 75%; e.g., Jeanneret et al., 2023; Vergauwe et al., 2023). In Experiment 4, we aimed to explore whether these differences in overall memory performance, rather than the nature of the memoranda per se, could explain the contrasting memory boost patterns between visuospatial and verbal experiments. To do so, our goal was to decrease the overall memory performance in a verbal experiment. We then aimed to test whether the cue-based and reward-based memory boost as assessed through the conventional comparisons would still be similar (as observed in the previous verbal experiments) or whether the cue-based memory boost would be larger than the reward-based boost (as seen in previous visuospatial experiments). To temper overall performance levels using verbal memoranda, we increased the number of black words to be remembered to 7 in Experiment 4. We again compared cue-based vs. reward-based prioritisation, and the prioritisation signal was always presented after encoding.

### Method

The preregistration for Experiment 4 can be found at https://osf.io/ja34c.

#### Participants and design

Our sample size was determined by the Bayesian sequential hypothesis testing procedure described in Supplementary Materials 1. A total of 120 (94 female, 25 male, 1 other; mean age = 23.4 years) undergraduate students were recruited from the University of Geneva and participated in exchange for partial course credit. All participants had normal or corrected-to-normal vision. No performance-based exclusions were applied. Thus, our final sample contains the data of 120 participants, including 60 participants in both the Cue and Reward groups.

We implemented a 2 × 2 design, with Prioritisation Mode (Cue vs. Reward) as between-subjects variable and Prioritisation Presence (Post-Prioritisation vs. No-Prioritisation) as within-subjects variable. Note that in comparison to Experiments 1–3, we no longer included distraction. In each group (Cue and Reward), Prioritisation Presence was implemented in blocks, and the order of the blocks was counterbalanced. In total, participants completed 294 experimental trials. As in Experiments 1–3, these trials were presented in blocks; this time of 147 trials each.^9^ Total experiment runtime, including consent and on-screen instructions, was about 60 min.

#### Materials and procedure

**Figure 8.**
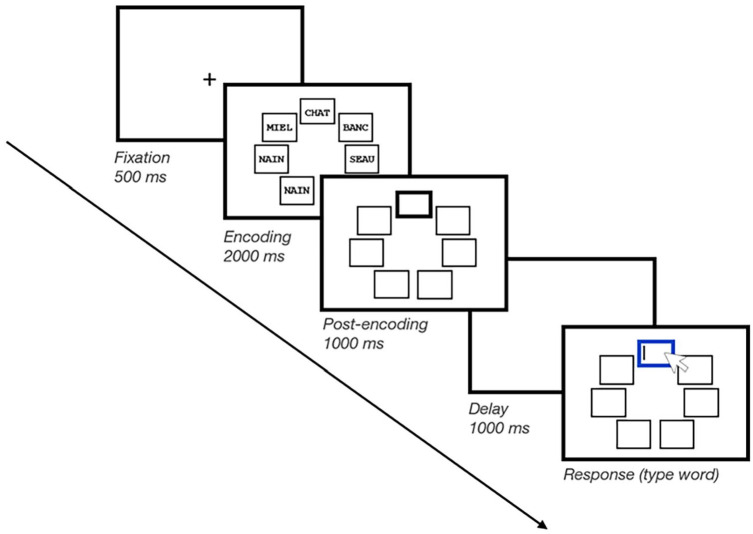
Schematic illustration of a trial in Experiment 4. Participants memorised seven short words, shown in seven different frames. These seven frames were also shown empty, after memory item presentation (i.e., post-encoding). On No-prioritisation trials, none of the frames was bolded; on Post-prioritisation trials, one of the seven frames was bolded after item presentation (as illustrated here). On all trials, after a brief retention interval that remained blank, memory was tested through cued recall.

We administered the task using E-Prime 3 (Psychology Software Tools) and used the same stimuli as Experiments 1–2: simple French words shown in black. The paradigm is most similar to that of Experiment 2, except for the following changes related to set size and the fact that we removed the distraction. As shown in Figure 8, we presented seven memory items in Experiment 4. These items were displayed simultaneously, for 2800 ms (400 ms per item, like in Experiments 1–2), at seven distinct spatial locations on an invisible heptagon, and each item was again enclosed by a frame. For the Post-*prioritisation* block, the spatial location of the black thick-bordered frame to induce prioritisation was again determined randomly on every trial, such that each memory item had equal probability of being prioritised, which was now about 14% because there were seven memory items on each trial.

As in Experiments 1–3, the prioritised item was always tested in the Cue group. In the Reward group, like in the previous experiments, the prioritised item was assigned a point reward equal to the set size number, and thus, correctly recalling the prioritised item now resulted in earning 7 points, whereas correctly recalling any of the other items resulted in earning 1 point (all seven items had equal probability of being tested). The 1000-ms post-encoding screen was always followed by a blank screen presented during 1000 ms. At the end of the trial, the test screen appeared, where one of the frames was highlighted with a thick blue border like in Experiments 1 and 2. Like in those experiments, the mouse cursor also appeared on screen, prompting participants to type the word they had memorised at the probed spatial location.

### Results and discussion

**Figure 9.**
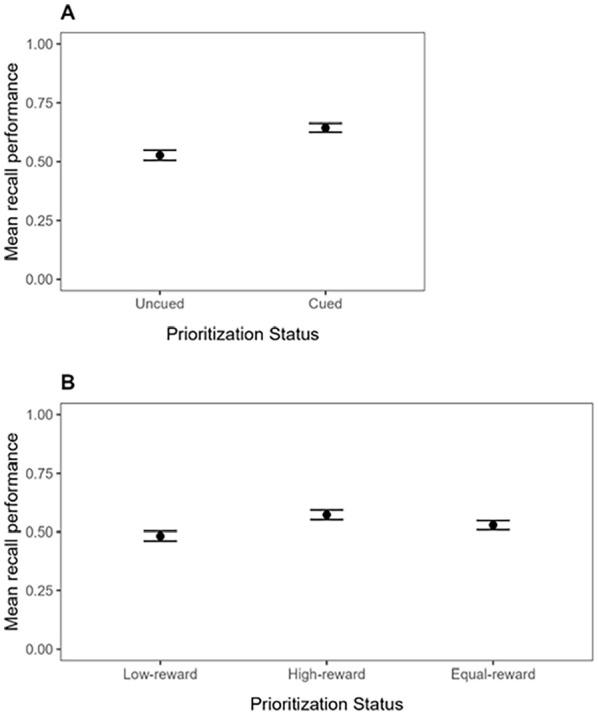
Mean recall performance in Experiment 4, as a function of Prioritisation Mode (Cue-based or Reward-based prioritisation) and Prioritisation Status. For Cue-based prioritisation (Panel A), Prioritisation Status includes Uncued or Cued. For Reward-based prioritisation (Panel B), Prioritisation Status includes Low-reward, High-reward, or Equal-reward. Error bars represent standard error of the mean.

Mean recall performance (proportion of correct responses to the test probes) for the Cue group and the Reward group is shown in Figure 9. In line with our preregistration, the data were analysed in two successive steps. While these steps are similar as those reported in Experiments 1–3, there are some differences, mainly related to the fact that Experiment 4 no longer included distraction or any manipulation related to distraction.

For the first analysis, we (1) extracted the data of the Cue group using the previously described conventional comparison (performance for prioritised items corresponded to performance observed for cued items and was extracted from Post-prioritisation trials, whereas performance for unprioritized items corresponded to performance observed for uncued items and was extracted from No-prioritisation trials). As before, this is referred to as “Cue group—conventional comparison.” Furthermore (2), we extracted the data of the Reward group using the previously described conventional comparison (performance for prioritised items corresponded to performance observed for high-rewarded items and was extracted from Post-prioritisation trials, and in the same way, performance for unprioritized items corresponded to performance observed for low-rewarded items and was extracted from Post-prioritisation trials). Like before, we call this “Reward group—conventional comparison.”

To compare the impact of prioritisation on memory performance between prioritisation modes, the data that were extracted as described for “Cue group—conventional comparison” and for “Reward group—conventional comparison” were analysed together using a Bayesian ANOVA of mean recall with Prioritisation Mode (Cue vs. Reward) as between-subjects variable and Prioritisation Status (Prioritised vs. Unprioritized). The best model of the data was the model with the two main effects (without the interaction). There was very strong evidence for the main effect of Prioritisation Status (BF_10_ = 7.58 × 10^16^), but only very weak evidence for the main effect of Prioritisation Mode (BF_10_ = 1.98), and the best model was only very weakly better than the full model including the interaction between Prioritisation Status and Prioritisation Mode (BF_01_ = 1.87 against the interaction). As such, it seems that the memory boost in both groups, assessed using conventional comparisons, should be considered rather similar. Accordingly, a preregistered one-sided *t*-test of the memory boosts (memory performance for Prioritised information minus memory performance for Unprioritized information), testing whether the memory boost is larger in the Cue group than in the Reward group, did not show evidence for that pattern (BF_01_ = 1.03). As seen in Figure 9, the differences in memory performance between cued and uncued items, on the one hand, and between high-reward and low-reward items on the other hand, did not appear very different (12 and 9 percentage points, respectively).

Next, for a second, more exploratory yet preregistered analysis, we extracted the data of the Reward group using the alternative comparison. Performance for prioritised items still corresponded to performance observed for high-rewarded items extracted from Post-prioritisation trials, but performance for unprioritized items corresponded now to performance observed for equally-rewarded items and was extracted from No-prioritisation trials. We then again compared the impact of prioritisation on memory performance between prioritisation modes, but this time by comparing the data of the “Cue group—conventional comparison” with the data of the “Reward group—alternative comparison.” These data were analysed together using a Bayesian ANOVA of mean recall with Prioritisation Mode (Cue vs. Reward) as between-subjects variable and Prioritisation Status (Prioritised vs. Unprioritized). The results were quite different from those observed when using the conventional comparison in the Reward group. Now, the best model was the full model including the main effects of Prioritisation Mode and Prioritisation Status as well as their interaction, and the evidence for the interaction was very strong (BF_10_ = 54.24).

A preregistered one-sided *t*-test of the memory boosts (memory performance for Prioritised information minus memory performance for Unprioritized information) now also showed very strong evidence for the memory boost being larger in the Cue group than in the Reward group (BF_10_ = 109). As can be seen in Figure 9, now that the comparison between cue and reward groups is more similar, the memory boost appears larger for cue-based prioritisation than for reward-based prioritisation (12 vs. 4 percentage points, respectively).

Together, the findings of these two analyses suggest that the memory boost for high-reward items as revealed by the conventional comparison (high-reward vs. low-reward items; 9 percentage points) reflects a combination of a genuine^10^ benefit for high-reward items (relative to equal items; 4 percentage points) and a cost for low-reward items (relative to equal items; 5 percentage points; see Table 2 for the relevant values). When focusing only on the benefit for prioritised information against baseline in both prioritisation modes, it appeared larger for information prioritised in a cue-based way.

Taken together, when using the conventional comparisons, we had previously observed a larger memory boost for cue-based prioritisation than for reward-based prioritisation across three visuospatial experiments that directly compared cue-based and reward-based prioritisation within a single paradigm (Jeanneret et al., 2023; Vergauwe et al., 2023). Using the same comparisons in the current study using verbal materials, we observed that the memory boost was rather similar across cue-based and reward-based prioritisation in Experiments 1–3. Here, in Experiment 4, still using the same comparisons as before, we again found a similar memory boost between cue-based and reward-based prioritisation with verbal materials. Thus, cue-based prioritisation did not result in a larger memory boost than reward-based prioritisation, even though we increased the set size to seven verbal items, which resulted in overall performance levels in verbal working memory comparable to those observed in the relevant studies in visuospatial working memory. However, when using the alternative comparison for reward-based prioritisation, it appears that the benefit of prioritised information against baseline is larger with cue-based prioritisation in verbal working memory. This will be further discussed in the general discussion.

## Experiment 5

In this final experiment, we aimed to explore whether the different patterns of memory boosts observed with cue-based vs. reward-based prioritisation in the visuospatial and verbal domains of working memory could be due to differences in response preparation and reconfiguration. When participants know in advance which item will be tested (as in cue-based prioritisation), they may begin preparing their response immediately, leading to an additional memory boost beyond the boost associated with directed attention. In the visuospatial analogue of our experiments (Experiment 2 of Vergauwe et al., 2023), participants were required to orally recall visually-presented shapes (see also Allen & Ueno, 2018; Hu et al., 2014, 2016), a task likely involving considerable response reconfiguration. This could explain why cue-based prioritisation produced a larger memory boost in these tasks (see the study by Myers et al., 2017, for the idea that response reconfiguration contributes to the retro-cue benefit in memory performance). In contrast, in our current verbal experiments, participants recalled visually-presented words by typing them, which may require less response reconfiguration, potentially limiting the memory boost associated with early response preparation in cue-based prioritisation.

To explore this possibility, Experiment 5 was designed to increase the demands of response reconfiguration in a verbal task. This experiment was similar to Experiment 1 (but without any distracting information during retention) but changed the response modality from typing to drawing. Participants memorised visually-presented shape nouns (e.g., “triangle,” “rectangle,” etc.) and recalled them by drawing the actual shapes at test. We reasoned that if response preparation enhances the memory boost in cue-based prioritisation, switching to drawing (which may require more response reconfiguration than typing) should reveal a larger memory boost for cue-based prioritisation, similar to what has been observed in visuospatial tasks. We again compared cue-based vs. reward-based prioritisation, with the prioritisation signal always presented after encoding.

### Method

The preregistration for Experiment 5 can be found at https://osf.io/qmzac.

#### Participants and design

Our sample size was determined by the Bayesian sequential hypothesis testing procedure described in Supplementary Materials 1. A total of 126 undergraduate students (107 female, 19 male, 0 other; mean age = 24 years) were recruited from the University of Geneva and participated in exchange for partial course credit. All participants had normal or corrected-to-normal vision. No performance-based exclusions were applied. However, one participant from the Cue group was excluded due to not having followed the instructions (submitted written rather than drawn responses). Thus, our final sample contains the data of 125 participants, including 63 and 62 participants in the Cue and Reward groups, respectively. We implemented a 2 × 2 design, with Prioritisation Mode (Cue vs. Reward) as the between-subjects variable and Prioritisation Presence (Post-prioritisation vs. No-prioritisation) as the within-subjects variable. Note that in contrast to Experiments 1–3, and like Experiment 4, we no longer included distraction.

#### Materials and procedure

We administered the task using E-Prime 3 (Psychology Software Tools). The stimuli were 10 French words describing familiar shapes: a triangle, rectangle, square, circle, rhombus, pentagon, arc, star, arrow, and heart. These 10 shape nouns were chosen based on a pilot test conducted in the lab using 15 different shapes. We asked five lab members to draw each of the different shapes and then selected the 10 most distinguishable shapes that were also most consistently drawn among all members.

**Figure 10.**
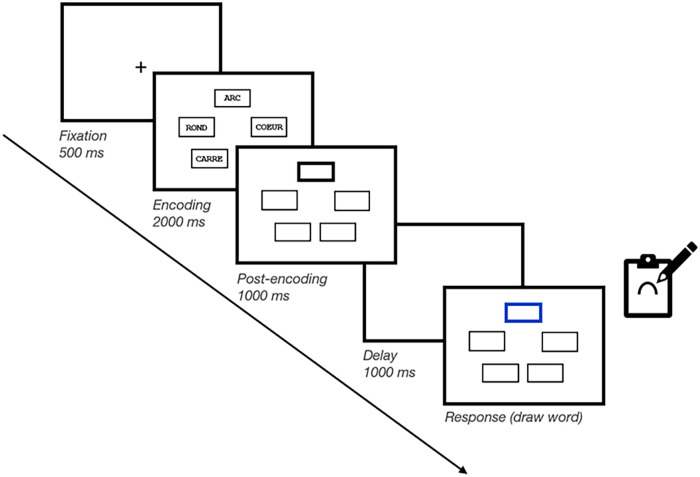
Schematic illustration of a trial in Experiment 5. Participants memorised five shape words, shown in five different frames. These five frames were also shown empty after memory item presentation (i.e., post-encoding). On No-prioritisation trials, none of the frames was bolded, on Post-prioritisation trials, one of the five frames was bolded after item presentation (as illustrated here). On all trials, after a brief retention interval that remained blank, memory was tested through cued recall. The corresponding shape had to be drawn using paper and pencil.

The paradigm is most similar to that of Experiment 2, except for the modification of the task response and removal of the distraction manipulations. As shown in Figure 10, participants memorised five shape words that appeared on screen simultaneously, at five distinct spatial locations. Depending on the experimental condition, participants may have had to prioritise one of these words via cue-based or reward-based prioritisation. At the end of the trial, they were probed to *draw* (rather than type) the shape word that had appeared at one of the spatial locations during encoding. Specifically, when the memory test screen appeared and one of the frames was highlighted with a thick blue border, the screen also displayed a question mark inside the highlighted frame, prompting participants to draw the shape they had memorised at the indicated location using a response sheet and a pencil. Each sheet contained 10 squares, allowing the participant to respond to 10 trials. Every 10 trials, a message was displayed on screen, asking the participant to turn the page. Participants pressed *Enter* using the keyboard as soon as they were done drawing the shape of a given trial. As in Experiments 1–4, even if they were not confident of their response, participants were encouraged to provide their best guess and to respond as quickly as possible. They were also allowed to correct their responses by erasing or crossing off their previous responses.

For the *Post-prioritisation* block, the location of the black bolded frame was randomly determined on every trial, such that each memory item had an equal probability of being prioritised (20%). In the Cue group, the prioritised item was always tested. In the Reward group, the single prioritised item was worth 5 points (all other items were still worth 1 point), and all five items had equal probability of being tested. In each group (Cue and Reward), Prioritisation Presence was implemented per block, and the order of the blocks was counterbalanced across participants. In total, participants completed 200 experimental trials. As in Experiments 1–4, these trials were presented in blocks; this time of 100 trials each. Task timings were identical to those implemented in Experiment 2. Total experiment runtime, including consent and on-screen instructions, was about 60 min.

### Results and discussion

**Figure 11.**
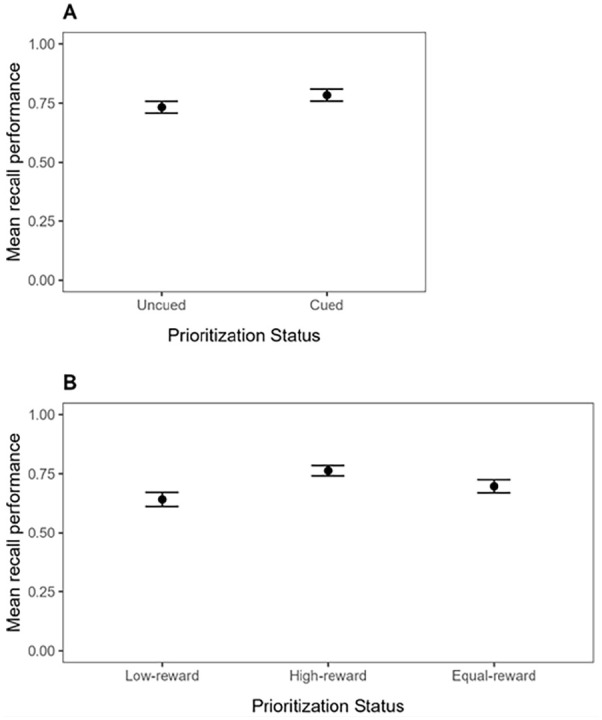
Mean recall performance in Experiment 5, as a function of Prioritisation Mode (Cue-based or Reward-based prioritisation) and Prioritisation Status. For Cue-based prioritisation (Panel A), Prioritisation Status includes Uncued or Cued. For Reward-based prioritisation (Panel B), Prioritisation Status includes Low-reward, High-reward, or Equal-reward. Error bars represent standard error of the mean.

Mean recall performance (proportion of correct responses to the test probes) for the Cue group and the Reward group is shown in Figure 11. In line with our preregistration, the data were analysed in the same two successive steps as the ones described for Experiment 4. For the first analysis, we extracted the data as described for the “Cue group—conventional comparison” and for “Reward group—conventional comparison” in the previous experiments. These were then analysed together using a Bayesian ANOVA of mean recall with Prioritisation Mode (Cue vs. Reward) as the between-subjects variable and Prioritisation Status (Prioritized vs. Unprioritized). The best model of the data was the full model, including the main effects of Prioritisation Mode and Prioritisation Status, as well as their interaction. There was some modest evidence for including the interaction, as removing the interaction rendered the model about three times worse (BF_10_ = 3.17). As can be seen in Figure 11, the boost was indeed somewhat different between cue-based and reward-based prioritisation. However, the difference was not in the expected direction, as the memory boost was more pronounced in the Reward group (high-reward vs. low-reward items; 12 percentage points) than in the Cue group (cued vs. uncued items; 5 percentage points). Accordingly, the preregistered one-sided *t*-test of the memory boosts (memory performance for Prioritised information minus memory performance for Unprioritized information) showed strong evidence against the memory boost being larger in the Cue group than in the Reward group (BF_01_ = 17.52).

Next, for the second, more exploratory but preregistered analysis, we extracted the data as described for the “Cue group—conventional comparison” and for “Reward group—alternative comparison” in the previous experiments and analysed these data together using a Bayesian ANOVA of mean recall with Prioritisation Mode (Cue vs. Reward) as the between-subjects variable and Prioritisation Status (Prioritised vs. Unprioritized). The results now showed a different best model, whereby only the main effect of Prioritisation Status is included (BF = 3875.63 over the null). There was some weak evidence against including the main effect of Prioritisation Mode (BF_01_ = 2.31), and the full model including the interaction term was 10.47 times worse than the best model. The preregistered one-sided *t*-test of the memory boosts showed again evidence against the memory boost being larger in the Cue group than in the Reward group (BF_01_ = 7.90). Thus, and as can be observed in Figure 11, the memory boost can be considered similar between the Reward group (7 percentage points) and the Cue group (5 percentage points), when prioritised items are compared against the baseline in both prioritisation conditions.

In line with what we observed in Experiment 4, the results of these two analyses suggest that the memory boost for high-reward items as evidenced by the conventional comparison (high-reward vs. low-reward items; 12 percentage points here) is a combination of a benefit for high-reward items (relative to equal-reward items; 7 percentage points here) and a cost for low-reward items (relative to equal-reward items; 5 percentage points here). In contrast to what we observed in Experiment 4, when we only compared the benefit portions of the memory boost, it was now found to be similar between information prioritised in a cue-based vs. reward-based way (5 and 7 percentage points, respectively).

Taken together, changing the response mode from typed recall to drawn recall at test did not result in a larger memory boost for cued information than for high-reward information, regardless of the comparison used to assess the memory boost. If anything, when using the conventional comparisons, the memory boost appeared to be somewhat larger for high-reward items than for cued items. Thus, neither increasing the set size in Experiment 4 nor increasing response reconfiguration demands in Experiment 5 resulted in the expected pattern of memory boosts in verbal working memory; the memory boost for prioritised information was not consistently larger for cue-based prioritisation than for reward-based prioritisation when using the conventional comparisons.

## General discussion

The current series of experiments aimed at investigating the consequences of directed attention in the verbal domain of working memory. This led to two main observations. First, we consistently observed a memory boost for prioritised information, regardless of whether cue-based or reward-based prioritisation was used. Overall, this boost can be considered rather similar in magnitude across cue-based and reward-based prioritisation. Second, verbal information did not suffer greatly from perceptual interference, even when presented auditorily. When perceptual interference *did* have a detrimental effect, it appeared to affect prioritized and unprioritized information similarly, with only weak evidence suggesting reduced distractor susceptibility for prioritised information and only when cued-based prioritisation was used. An overview of these findings can be found in Table 3. The implications are discussed below.

**Table 3.** Overview of findings, across Experiments 1–5.

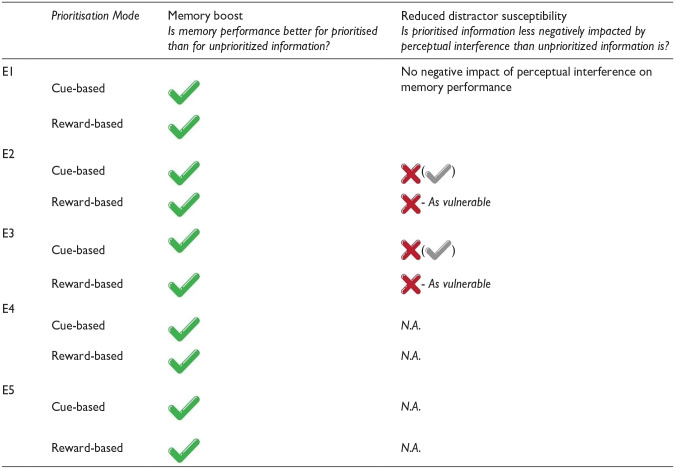

*Note.* Green symbol 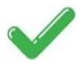 indicates “consistently yes”; red symbol 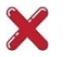 indicates “consistently no”; grey symbol 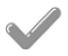 indicates “yes, but not very consistently or convincingly so, or only in un-preregistered analyses.” Note that 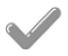 was only added in the table when the preregistered BANOVA showed no evidence, but the un-preregistered *t*-tests provided evidence (1) *for* a negative impact on prioritised items as well as (2) *against* a negative impact on unprioritized items. If either of these two relevant *t*-tests was inconclusive (i.e., BF < 3), we did not consider the evidence convincing enough to suggest reduced distractor susceptibility for prioritised information. In such cases, we concluded that both prioritised and unprioritized items were equally vulnerable to distraction.

### Better memory for prioritised information in verbal working memory

Only a few studies have examined the enhancement of memory performance by directing attention during retention in verbal working memory. In line with these studies, we consistently observed better memory performance for prioritised information than for unprioritized information, both under cue-based and reward-based prioritisation. In previous research using cue-based prioritisation, a memory boost had been observed for cued letters that were presented simultaneously, quite fast and in an array (125–250 ms per letter in Krefeld-Schwalb, 2018; 150 ms per letter in Shepherdson et al., 2018). However, Shepherdson and colleagues (2018) observed no memory boost for cued verbal items when they were presented more slowly (500 ms per word) and sequentially. The fast presentation times used in the experiments presenting verbal information simultaneously may have encouraged visual encoding of the information, casting some doubt on whether cue-based prioritisation can lead to a memory boost of verbal representations. In our experiments, however, we repeatedly observed a memory boost for words that were shown simultaneously in an array but more slowly than in previous studies using arrays (400–500 ms per word). This suggests that the mode of presentation (simultaneous vs. sequential) may be critical in determining whether or not cue-based prioritisation leads to a memory boost for verbal information, rather than presentation duration per se. Overall, the fact that we consistently observed a memory boost in our experiments confirms that verbal information *can* benefit from cue-based prioritisation during retention.

Previous research using reward-based prioritisation of verbal materials had only induced the priority before or during encoding (Atkinson et al., 2021; Sandry et al., 2014, 2020). Our experiments are the first to show that reward-based prioritisation of verbal information after encoding leads to a memory boost as well. In Experiment 1, we also observed that the improvement of memory performance was similar whether the reward-based priority was induced before or after the information was encoded into working memory. Moreover, in our experiments, verbal memory materials were presented visually, in arrays. This approach is consistent with previous experiments on prioritisation of visuospatial materials and on cue-based prioritisation of verbal materials. However, it differs from previous studies on reward-based prioritisation of verbal materials. In those studies, verbal memory materials were always presented sequentially, either visually (Sandry et al., 2014, 2020) or auditorily (Atkinson et al., 2021). Collectively, our current experiments and those reported by Atkinson and colleagues (2021) and by Sandry and colleagues (2014, 2020) demonstrate a consistent and robust memory boost for verbal materials due to reward-based prioritisation, across a wide range of methodological differences, including simultaneous vs. sequential presentation of the memoranda. This last observation diverges from the pattern that is emerging from studies using cue-based prioritisation of verbal materials, where the memory boost appears to be limited to simultaneously presented information. However, to draw firm conclusions, future research should focus on a more direct comparison of the effects of prioritisation on verbal information when presented simultaneously vs. sequentially.

To date, no study has directly compared the memory boost between cue-based and reward-based prioritisation of verbal materials. Thus, it has remained unknown whether the boost for verbal materials, similar to findings using retrospective prioritisation of visuospatial materials, is smaller and less consistent with reward-based than with cue-based prioritisation. Here, we directly compared these memory boosts and found consistent evidence for a memory boost for both types of prioritisation of verbal materials. Moreover, these boosts were remarkably similar when using conventional comparisons (i.e., the comparisons used in most cue-based prioritisation studies and in early reward-based studies). In these comparisons, the memory boost associated with cue-based prioritisation is calculated by comparing memory performance between cued and uncued items, with uncued items coming from trials without priority induction. The memory boost associated with reward-based prioritisation, on the other hand, is calculated by comparing memory performance between high-reward items and low-reward items, with low-reward items coming from trials *with* priority induction. Using these comparisons, we found that the memory boost for cue-based prioritisation was very similar in magnitude as the memory boost for reward-based prioritisation (14 vs. 15 percentage points, respectively), when we only considered Experiments 1–3. Taking now *all five* experiments into account, these memory boosts remain remarkably similar, with memory boosts of 12 vs. 13 percentage points, for cue-based and reward-based prioritisation, respectively. Overall, when using the comparisons commonly used in most cue-based studies and in early reward-based studies, our data indicate that memory performance for simultaneously presented verbal memory materials benefits consistently from cue-based and reward-based prioritisation and to the same extent.

However, we were also able to examine the memory boost for high-reward items in a different way, that is more similar to what is done in more recent reward-based studies. In these studies, memory performance for a prioritised item is compared to memory performance for unprioritized items coming from trials without priority induction. Using this alternative comparison (i.e., high-reward items vs. equal-reward items), we still observed clear evidence for a memory boost across all our experiments. However, as can be seen in Table 2, these memory boosts were smaller than those revealed by the conventional comparison within the Reward groups. Across our five experiments, we observed memory boosts of 13 vs. 7 percentage points, for the conventional vs. alternative comparisons of reward-based prioritisation, respectively. This indicates that, for reward-based prioritisation, the conventional comparison reflects a combination of a genuine benefit in performance for high-reward items (relative to baseline, i.e., to equal-reward items) and a cost in performance for low-reward items (again relative to baseline, i.e., to equal-reward items; see the study by Allen et al., 2024, for a similar conclusion).

Across our experiments, the genuine benefit for high-reward items and the cost for low-reward items appear to be similar in size, with an average of 7 percentage points for the benefit and of 6 percentage points for the cost. It is worth noting that the conventional comparison for cue-based prioritisation can only reflect a genuine benefit for cued items, as we compared memory performance directly to uncued items from baseline trials. Comparing now only the genuine benefit for prioritised information (against baseline) between cue-based and reward-based prioritisation across our five experiments, it is larger for cue-based prioritisation (12 percentage points) than for reward-based prioritisation (7 percentage points). However, this was not consistently the case, as the genuine benefit for cued information was larger in only three out of the five experiments.

Taken together, while the memory boost as often calculated in cue-based experiments and in early reward-based experiments was found to be similar across cue-based and reward-based prioritisation, the genuine benefit for prioritised information appears sometimes a bit larger with cue-based prioritisation than with reward-based prioritisation. The fact that, for reward-based prioritisation, this benefit was accompanied by a cost for low-reward items indicates that reward-based prioritisation during retention might have resulted in resources being allocated to the high-reward item and thus diverted away from low-reward items. One way to do so would be through biased attentional refreshing, whereby high-reward items are refreshed more during retention, as suggested by Atkinson et al. (2022).

Having discussed our main observations on the memory boost for prioritised verbal memory materials, we now compare these findings with those previously observed with visuospatial materials. Two points are worth mentioning. First, evidence for a memory boost was consistently found for both cue-based and reward-based prioritisation in our verbal experiments, but some published experiments indicate that the cue-based memory boost may be absent for sequentially presented verbal materials (e.g., Shepherdson et al., 2018). In the visuospatial domain, evidence for a memory boost has been observed for both retrospective cue-based and reward-based prioritisation, but this boost was less consistently observed with reward-based prioritisation (e.g., Hautekiet et al., 2024; Jeanneret et al., 2023; Zhang & Lewis-Peacock, 2023b). Moreover, a cue-based memory boost has also been observed for sequentially presented visuospatial information (Hautekiet et al., 2023). It thus appears that both verbal and visuospatial information can benefit from both prioritisation modes, but the boundary conditions may differ between domains; boundary conditions are mainly observed for cue-based prioritisation in verbal working memory, while they are mainly observed for reward-based prioritisation in visuospatial working memory. Future studies should examine these differences in more detail.

Second, with retrospective prioritisation of visuospatial materials, the conventional memory boost is often much larger with cue-based prioritisation than with reward-based prioritisation (e.g., 23 vs. 8 percentage points, respectively, across the experiments reported in the study by Jeanneret et al., 2023, and in Vergauwe et al., 2023). Here, with verbal materials, however, conventional comparisons revealed a similar memory boost across these prioritisation modes (12–13 percentage points). In our Experiments 4 and 5, we aimed to test whether this difference between verbal and visuospatial experiments could be accounted for by either overall performance levels or response configuration demands. Manipulating these variables did not, however, result in a larger cue-based memory boost, compared to the reward-based memory boost, using the conventional comparisons. Comparing the memory boosts using more equivalent comparisons between cue-based and reward-based prioritisation showed that the memory boost, relative to baseline, is sometimes larger for cued items than for high-reward items in our verbal experiments, but not always. Overall, whereas cue-based prioritisation results in more consistent and consistently larger memory boosts than reward-based prioritisation in visuospatial working memory, the memory boosts associated with cue-based and reward-based prioritisation appear much more similar in verbal working memory. The overall pattern is consistent with the operation of a domain-general focus of attention in working memory (see also recent findings of Roe et al., 2024) along with an additional process that specifically boosts cued visuospatial information. More research is needed to uncover the specific nature of this extra process that appears domain-specific in nature.

### Not much evidence for (reduced) distractor vulnerability in verbal working memory

A second benefit that is often associated with directed attention during retention is reduced distractor susceptibility, whereby prioritised information is less affected by perceptual interference than unprioritized information. To date, this has not been studied yet in verbal working memory. In our experiments, we did not observe much evidence for reduced distractor susceptibility. In Experiment 1, in which the distraction was very similar to what had been used in relevant visuospatial studies (1 suffix item, presented visually, in the centre of the screen), we did not find any disruptive effect of the perceptual interference at all. After modifying the experiment in different ways, we *did* observe a negative effect of distraction on memory performance in Experiment 2 (1 suffix item, presented visually, in all memory locations, as well as auditorily) and Experiment 3 (1 suffix item, presented visually, in all memory locations, while using coloured words as memoranda instead of black words). Importantly, however, our preregistered analyses showed that our data did not strongly support the hypothesis of distractor susceptibility being modulated by whether the information was prioritised or not. Specifically, for cue-based prioritisation, there was no evidence for the predicted interaction that should emerge if prioritised information is impacted differently by distraction compared to unprioritized information. However, un-preregistered follow-up analyses did show some evidence for prioritised information being less disrupted by perceptual interference than unprioritized information. Therefore, we believe there is *some* evidence for reduced distractor susceptibility with cue-based prioritisation. However, the evidence is far from strong.

For reward-based prioritisation, there was evidence *against* the predicted interaction between the status of an item (prioritised vs. unprioritized) and the presence of perceptual interference in both Experiments 2 and 3. Furthermore, the un-preregistered follow-up analyses did not show clear evidence for prioritised information being less affected by perceptual interference than unprioritized information. Instead, both prioritised and unprioritized information appeared vulnerable and to a similar extent. Thus, taken together, across prioritisation modes, the negative impact of perceptual interference was not very drastic nor was it very robust in our verbal experiments. Moreover, when a negative impact was observed, the evidence for it being modulated by whether or not the memory item had been prioritised during retention was modest at best.

It is worth noting that our observations in verbal working memory sharply contrast with the idea that prioritising information during retention makes it highly available and accessible yet also “highly fragile and vulnerable to perceptual interference” (Baddeley et al., 2021, p. 26). A strict interpretation of our data indicates that prioritised and unprioritized information are equally vulnerable to perceptual interference. A more lenient interpretation of our data suggests that prioritised and unprioritized information are equally vulnerable to perceptual interference under reward-based prioritisation, while prioritised information tends to be less vulnerable to perceptual interference under cue-based prioritisation. We do not believe that there is anything in our data suggesting that prioritised information is *more* vulnerable to perceptual interference than unprioritized information. Since our experiments were the first to examine the impact of distraction for prioritised and unprioritized information in verbal working memory, we believe there is currently no evidence for a highly fragile state in verbal working memory.

One could argue that, by aiming to stay close to the visuospatial counterpart of our experiments, our manipulation of perceptual interference was not ideal. Indeed, staying close to these previous studies, two of our current experiments presented purely visual distraction during retention. The pure visual nature of the interfering item may explain why the evidence was not consistently strong for a negative impact on memory performance. If verbal representations are closely related to speech and to the auditory modality (e.g., Hitch & Baddeley, 1976; see also Atkinson et al., 2021), then verbal representations held in working memory should not be impacted drastically by visually-presented distractions. This should be all the more the case given that we presented the information quite slowly and did not impose articulatory suppression, as to facilitate verbal encoding of the memoranda in our experiments. We acknowledge that introducing purely visual interference might not be the strongest possible manipulation to examine distractor susceptibility in verbal working memory. However, in Experiment 2, we introduced auditory distraction during retention as well, and yet, the same overall pattern of results was observed. This indicates that even with more optimally designed distraction, the negative impact on memory performance is not drastic, and the evidence for a difference in impact between prioritised and unprioritized information is weak.

There is another reason why one could argue that, by aiming to stay close to the visuospatial counterpart of our experiments, the current experiments were not optimally designed to detect a negative impact of perceptual interference. In our experiments, to-be-remembered words were presented simultaneously, whereas the classic suffix effect has predominantly been shown for sequentially presented verbal items (e.g., Crowder, 1967; Crowder & Morton, 1969; Greene et al., 1988). One could argue that suffix effects are likely to be small when verbal materials are presented simultaneously, limiting hence the sensitivity of our experiments to detect any modulation of perceptual interference as a function of prioritisation.^11^ Four points are relevant here. First, using simultaneous presentation is not uncommon when studying verbal working memory (e.g., Depoorter & Vandierendonck, 2009; Ferber & Danckert, 2006; Ma et al., 2017; Peterson et al., 2019; Sheu et al., 2019; Smith et al., 1998; Souza et al., 2018). Second, using sequential presentation of verbal information would confound the nature of the memory materials with presentation mode when comparing verbal and visuospatial experiments. Third, a recent study indicates that cue-based prioritisation may not lead to a memory boost for sequentially presented verbal memory materials (Shepherdson et al., 2018). Finally, in some of our experiments, there was evidence for a negative impact of distraction, but the data indicated that the effect was not modulated by the status of the information (prioritised vs. unprioritized). It is possible that sequentially presented verbal memory materials may be more vulnerable to perceptual interference, and this should be explored in future studies. It would be useful to also include auditory presentation of verbal memory materials in those studies, to further reduce the potential involvement of visual memory codes of the verbal memory materials. Moreover, it could be useful to also include different reward patterns, since prioritising more than one item may result in a clearer pattern of distractor vulnerability varying as a function of prioritisation status (Allen & Ueno, 2018).

In visuospatial working memory, the effect of prioritisation on distractor susceptibility appeared to depend on the prioritisation mode. With cue-based prioritisation, prioritised information is typically less affected by perceptual interference than unprioritized information is. With reward-based prioritisation, however, reduced susceptibility is not observed; prioritised information is either found to be as vulnerable to perceptual interference as unprioritized information is or even *more* so. At first sight, our findings in verbal working memory may seem quite different. Indeed, we (1) did not find verbal memory to be greatly impacted by perceptual interference, (2) only found some weak evidence for reduced distractor susceptibility with cue-based prioritisation, and (3) did not find distractor susceptibility to be affected by prioritisation status with reward-based prioritisation. However, a closer look indicates that the differences between the verbal and visuospatial domains may be rather limited when it comes to distractor susceptibility, for several reasons.

First, the evidence for a negative impact of perceptual interference in visuospatial working memory is less consistent than we initially thought. For example, some of our recent studies did find an overall negative impact on visuospatial memory performance (e.g., Hautekiet et al., 2023; Vergauwe et al., 2023), but we have another recent series of experiments, which varied widely in methodological details, where we did not observe a detrimental effect of perceptual interference on visuospatial memory performance (Hautekiet et al., 2024, see also the study by Zhang & Lewis-Peacock, 2023b, for similar recent findings). Second, although reduced distractor susceptibility has often been observed with cue-based prioritisation of visuospatial information, this finding also seems less consistent than we initially assumed. For instance, in the study by Vergauwe et al. (2023), we did find evidence for the predicted interaction under cue-based prioritisation, whereas Hautekiet et al. (2023) did not.

Thus, overall, the current findings in verbal working memory are somewhat inconsistent with the pattern we initially thought to emerge from the literature on visuospatial working memory. However, as we have described, based on a set of recent studies, it appears that the pattern in visuospatial working memory itself is less conclusive and consistent than we had assumed at the start of the current series of experiments. Unlike the memory boost, which exhibits a clear and coherent pattern, a consistent pattern is not really observed for distractor susceptibility. Therefore, we refrain from firm conclusions about whether and how prioritisation influences distractor susceptibility, and whether this varies consistently across different prioritisation modes and domains.

### Conclusion and relation to the evolution of the multiple-component model

Overall, we observed strong and consistent evidence for one of the assumed consequences of directed attention during retention in working memory, with better memory performance for verbal materials that were prioritised after encoding, regardless of whether prioritisation was cue-based or reward-based. This finding is mostly consistent with observations in visuospatial working memory and may reflect the operation of domain-general attention in working memory. Even though the multiple-component model is mostly known for its emphasis on domain-specific aspects of working memory, domain-general resources were included in the original versions of the model (see the study by Baddeley & Hitch, 1974; Hitch & Baddeley, 1976). The domain-general aspect of the model is situated at the central level, which Hitch and Baddeley (1976) referred to as “central WM executive”. The theoretical concepts of prioritisation, directing attention, and the focus of attention have been proposed as inherently linked to the central, domain-general part of working memory within the model. As such, our observation that several aspects related to the consequences of directed attention during retention extend from the visuospatial domain to the verbal domain of working memory is consistent with recent versions of the model. We also observed some aspects that differ between verbal and visuospatial materials, which could be accounted for by some of the domain-specific mechanisms proposed in the model.

However, the most recent updates of the model proposed the central focus of attention as a state in which information is particularly fragile and vulnerable to perceptual interference (e.g., Baddeley et al., 2021). Despite this, findings on distractor susceptibility vary considerably across studies, and our current data do not support this idea. Recent studies in visuospatial working memory such as those by Hautekiet et al. (2024) and Vergauwe et al. (2023) are also inconsistent with this idea. Instead, we sometimes find that information receiving focused attention during retention is protected, but often it is just as vulnerable as information held outside the focus of attention.

In summary, our findings are consistent with the idea of a central, domain-general focus of attention in working memory that boosts memory performance for prioritised items, but they also suggest the operation of additional processes or mechanisms. Finally, our results call for a more nuanced view whereby focused attention does not consistently render information more (or less) susceptible to perceptual interference.

## Supplemental Material

sj-docx-1-qjp-10.1177_17470218241299918 – Supplemental material for What are the benefits of directed attention within verbal working memory? Supplemental material, sj-docx-1-qjp-10.1177_17470218241299918 for What are the benefits of directed attention within verbal working memory? by Stéphanie Jeanneret, Evie Vergauwe, Caro Hautekiet and Naomi Langerock in Quarterly Journal of Experimental Psychology

## Notes

1. All secondary tasks used by Krefeld-Schwalb (2018) are different from the perceptual interference conditions that are typically used in visuospatial working memory experiments to examine distractor susceptibility of prioritised information because these secondary tasks all require active processing as well as decision-making. Among the three secondary tasks used by Krefeld-Schwalb (2018), the *perception* task is the most similar to standard perceptual interference conditions. In this task, participants judged the symmetry of visually-presented letters. This is different from the *retention* and *imagery* tasks, where participants judged the symmetry of letters that were recently presented, either visually or auditorily, but were not visible when participants had to make the decision.

2. The investigation of the impact of prioritisation during retention requires, strictly speaking, only the presentation of the prioritisation signal after encoding, i.e., when all information has been encoded and can be assumed to be represented in working memory. However, reward-based prioritisation studies using verbal material have only induced prioritisation either before or during encoding. To limit the number of experimental conditions in our experiment, we did not include all three possibilities. Instead, we only included the two time points that have been used most frequently in prioritisation studies in general: before and after encoding.

3. In some cases (<5, and only for Experiments 2–3), participants from the Cue and Reward groups were tested in the same testing room for practical purposes.

4. The corresponding variable is called “freqfilms2” and reflects the frequency of the word according to the subtitles corpus.

5. Note that this comparison was referred to as “Cue group—Classic comparison” in our preregistration.

6. Note that this comparison was referred to as “Reward group—Classic comparison” in our preregistration.

7. Audio recordings were created using “Lea” on https://ttsmp3.com/text-to-speech/French/.

8. Although performance was high in some conditions, this cannot explain our main findings. In Experiment 1, performance was very high for cued items, but only when the cue was presented before encoding. There was no difference in results depending on whether priority was induced before or after encoding; there was a memory boost due to the cue in both cases, without any negative impact of distraction. In Experiment 3, memory performance was again high for cued items. This was one of the conditions showing some weak evidence for protection. However, similar weak evidence for protection was found in the same condition in Experiment 2, where memory performance for cued items was much lower. Therefore, our conclusions are not meaningfully impacted by a ceiling effect.

9. In Experiment 4, we aimed to match the absolute number of trials testing high-reward items to that in Experiments 1–2, where there were 20 such trials using a set size of 5. However, we adjusted for the fact that in the Reward group of Experiment 4, the likelihood of any item being tested changed from 1 out of 5 (as in Experiments 1–2) to 1 out of 7. To achieve this, we used blocks of 147 trials, which allowed us to test 21 high-reward items.

10. We use the word “genuine” to emphasise that the observed memory benefit for high-reward items is a true positive effect relative to a neutral baseline (i.e., equal-reward items), and not the result of comparing high-reward to low-reward items, with the latter possibly suffering from a performance cost.

11. We thank an anonymous reviewer for this suggestion.
