# Supplementary material for: What are the benefits of directed attention within verbal working memory?
Source: Q J Exp Psychol (Hove). 2024 Dec 10;78(2):337–69. doi: 10.1177/17470218241299918 (PMC11783979; doi:10.1177/17470218241299918)
Supplement: sj-docx-1-qjp-10.1177_17470218241299918 – Supplemental material for What are the benefits of directed attention within verbal working memory? [file sj-docx-1-qjp-10.1177_17470218241299918.docx]

Supplementary materials for:

**What are the benefits of directed attention within verbal working memory?**

Stéphanie Jeanneret*, Evie Vergauwe*, Caro Hautekiet, and Naomi Langerock

University of Geneva

Switzerland

**Joint first-authorship*

**Word count:** 20’046

**Running head**: Attention and distraction in verbal WM

**Author Note**

Evie Vergauwe and Stéphanie Jeanneret share first authorship. This work was supported by the Swiss National Science Foundation [Grant number PCEFP1_181141 to Evie Vergauwe]. Correspondence should be sent to Evie Vergauwe and Stéphanie Jeanneret, Université de Genève, Faculté de Psychologie et des Sciences de l'Éducation, 40 bd du Pont d’Arve, 1211 Genève 4, Switzerland, [Evie.Vergauwe@unige.ch](mailto:Evie.Vergauwe@unige.ch) and [Stephanie.Jeanneret@uth.tmc.edu](mailto:Stephanie.Jeanneret@uth.tmc.edu).

**Conflict of interest**

The authors have no conflict of interest to declare.

**Data availability statement**

All data is available on Open Science Framework: <https://osf.io/jpqvn/>

**Supplementary materials 1: Additional information regarding the Bayesian sequential hypothesis testing procedure and the resulting sample sizes**

**Experiment 1**

We planned to collect a first batch of 60 participants (i.e., 30 participants in each group: Cue and Reward) and to use Bayesian sequential hypothesis testing to assess the data’s evidence for or against the interaction of interest in each group and subsequently, determine whether a second batch was needed. The interaction of interest concerned the interaction between the prioritization status of an item and the presence of distraction (i.e., the interaction between Prioritization Timepoint and Distraction in the Cue group, and the interaction between Prioritization Status and Distraction in the Reward group, see analyses below for more details). If the BF had been lower than 10 for or against the interaction of interest in the Cue group, a second batch of 30 participants was going to be tested in this group. If the BF had been lower than 10 for or against the interaction of interest in the Reward group, a second batch of 30 participants was going to be tested in this group. Thus, in each group, testing was planned to either stop after the first batch of 30 participants because the Bayes Factor of interest reached 10, resulting in a final sample size of 30 participants for a given group, or stop after the second batch of 30 participants because the Bayes Factor of interest did not reach 10 after the first batch, resulting in a final sample size of 60 participants for a given group. A total of 62 participants were tested in total.

**Experiment 2**

We used the same sampling plan as in Experiment 1. The interaction of interest was again concerned with the interaction between the prioritization status of an item and the presence of distraction (i.e., the interaction between Prioritization Presence and Distraction in the Cue group, and the interaction between Prioritization Status and Distraction in the Reward group, see analyses below for more details). Doing so, we tested 63 participants in the first batch (33 and 30 participants in the Cue and Reward groups, respectively). Given inconclusive results in the first batch, we collected a second batch of participants: 62 participants were tested (30 and 32 participants in the Cue and Reward groups, respectively).

**Experiment 3**

We used the same sampling plan as in Experiments 1 and 2. The interaction of interest was again concerned with the interaction between the prioritization status of an item and the presence of distraction (i.e., the interaction between Prioritization Presence and Distraction in the Cue group, and the interaction between Prioritization Status and Distraction in the Reward group, see analyses below for more details). In doing so, we tested 63 participants in the first batch (33 and 30 participants in the Cue and Reward groups, respectively). Given inconclusive results in the first batch, we collected a second batch of participants: 81 participants were tested (43 and 38 participants in the Cue and Reward groups, respectively).

**Experiment 4**

We used a similar sampling plan as in Experiments 1-3. However, the interaction of interest was now the interaction between Prioritization Mode (Cue vs. Reward) and Prioritization Status (Prioritized vs. Unprioritized). We had planned to test a first batch of 60 participants (30 participants in both the Cue and Reward groups) and, if the BF was lower than 10 for or against the interaction of interest, a second batch of 30 participants would be tested in both groups (i.e., an additional 30 participants in both the Cue and Reward groups). Given inconclusive results in the first batch (30 participants in both groups), we collected a second batch of 60 participants (30 participants in both groups).

**Experiment 5**

We use the same sampling plan as in Experiment 4, with the same interaction of interest. In the first batch, we tested 65 participants (33 and 32 participants in the Cue and Reward groups, respectively). Given inconclusive results in the first batch, we collected a second batch of participants (61 in total, including 31 and 30 participants in the Cue and Reward groups, respectively).

**Information concerning all experiments**

Overall, due to the need to compensate for planned participants who did not show up, scheduling challenges led us to test more participants than the initially planned 30 in several of the batches: batch 1 of the Reward group in Experiment 1 (32 participants), batch 1 of the Cue group in Experiment 2 (33 participants), batch 2 of the Reward group in Experiment 2 (32 participants), batches 1 and 2 of the Cue group in Experiment 3 (33 and 43 participants, respectively), batch 2 of the Reward group in Experiment 3 (38 participants), batches 1 and 2 of the Cue group in Experiment 5 (33 and 31 participants, respectively), and batch 1 of the Reward group in Experiment 5 (32 participants).

**Supplementary materials 2: Supplementary Analyses Experiment 1**

**Cue group – conventional comparison.** The impact of cue-based prioritization was also examined for the different timepoints separately. To examine the impact of a cue presented before encoding, we performed a repeated-measures BANOVA with Prioritization Status (Cued vs. Uncued, extracted from Pre-prioritization and No-prioritization trials, respectively) and Distraction (Suffix vs. No suffix) as within-subjects variables. To examine the impact of a cue presented after encoding, we performed a repeated-measures BANOVA with Prioritization Status (Cued vs. Uncued, extracted from Post-prioritization and No-prioritization trials, respectively) and Distraction (Suffix vs. No suffix) as within-subjects variables. In both analyses, the Prioritization Status-only model was found to be the best model of the data (BFs of 5.39x10^15^ and 8815 against the null, respectively). In both cases, there was evidence against the main effect of Distraction (BF_01_ = 5.11 and BF_01_ = 3.70, respectively), and the full model including the interaction of interest was about 15 times worse than the best model (BFs of 17.40 and 14.32, respectively).

**Reward group – conventional comparison.** The impact of reward-based prioritization was also examined for the different timepoints separately. To examine the impact of a reward presented before encoding, we performed a repeated-measures BANOVA with Prioritization Status (High-reward vs. Low-reward, both extracted from Pre-prioritization trials) and Distraction (Suffix vs. No suffix) as within-subjects variables. To examine the impact of a reward presented after encoding, we performed a repeated-measures BANOVA with Prioritization Status (High-reward vs. Low-reward, both extracted from Post-prioritization trials) and Distraction (Suffix vs. No suffix) as within-subjects variables. In both analyses, the Prioritization Status-only model was found to be the best model (BFs of 226 and 1.26x10^6^ against the null, respectively), and there was evidence against the main effect of Distraction (BF_01_ = 5.26 and BF_01_ = 4.10, respectively). The full model including the interaction of interest was considerably worse than the best model (BFs of 8.29 and 15.78, respectively).

**Reward group – alternative comparison.** For this comparison as well, the impact of reward-based prioritization was also examined for the different timepoints separately. To examine the impact of a reward presented before encoding, we performed a repeated-measures BANOVA with Prioritization Status (High-reward vs. Equal-reward, extracted from Pre-prioritization and No-prioritization trials, respectively) and Distraction (Suffix vs. No suffix) as within-subjects variables. To examine the impact of a reward presented after encoding, we performed a repeated-measures BANOVA with Prioritization Status (High-reward vs. Equal-reward, extracted from Post-prioritization and No-prioritization trials, respectively) and Distraction (Suffix vs. No suffix) as within-subjects variables. In both analyses, the Prioritization Status-only model was the best model (BFs of 26.22 and 193.74 against the null, respectively). In both cases, there was evidence against the main effect of Distraction (BF_01_ = 3.59 and BF_01_ = 5.38, respectively), and the full model including the interaction of interest was substantially worse than the best model (BFs of 12.35 and 18.18, respectively).

**Vulnerability scores.** The conventional comparisons were used to calculate vulnerability scores. In particular, in the Cue group, memory performance for prioritized items corresponded to the average memory performance for the cued items across Pre-prioritization and Post-prioritization trials; memory performance for unprioritized items corresponded to memory performance for the uncued items on No-Prioritization trials. In the Reward group, memory performance for prioritized items corresponded to the average memory performance for the high-reward item across Pre-prioritization and Post-prioritization trials; similarly, memory performance for unprioritized items corresponded to the average memory performance for the low-reward items across Pre-prioritization and Post-prioritization trials.

In a set of unpreregistered, one-sided t-tests, we assessed, for each vulnerability score shown in Figure 3, Panel A, of the manuscript whether there was evidence in the data for the score being larger than 0, i.e., for the information being vulnerable. This confirmed that there was no clear evidence in the data for a negative impact of interference on memory performance; BF_01_ = 4.91 for cued items, BF_01_ = 1.82 for uncued items, BF_01_ = 6.91 for high-reward items, and BF_10_ = 1.65 for low-reward items.

**Supplementary materials 3: Additional information on the memory boost observed in visuospatial studies comparing** **cue-based and reward-based prioritization**

In visuospatial working memory, the memory boost is often larger and more consistent for cue-based than for reward-based prioritization in studies directly comparing cue-based and reward-based prioritization. For example, in Jeanneret et al. (2023), we investigated the memory boost for visual objects, comparing cue-based and reward-based prioritization within the same paradigm (only post-encoding). This was one of the first studies that used both cue-based and reward-based prioritization within the same paradigm. Informed by previous work using either cue-based or reward-based prioritization in separate studies, we examined the memory boost for prioritized information as had been done in the relevant studies. Thus, in Jeanneret et al. (2023), we used what we have referred to here as conventional comparisons. In two experiments, we observed a memory boost of 21 percentage points for cue-based prioritization. In contrast, for reward-based prioritization, the memory boosts had a magnitude of only 5 and 7 percentage points in Experiments 1 and 2, respectively. Similarly, in Vergauwe et al. (2023), we found a memory boost of 26 percentage points for cue-based prioritization and a smaller memory boost of 13 percentage points for reward-based prioritization, using the conventional comparisons (only considering post-encoding prioritization). Across these three experiments, using the conventional comparisons, the memory boost for cue-based prioritization appeared almost three times greater in magnitude than the memory boost for reward-based prioritization (23% vs. 8%, respectively).
